# The potential role of *MGMT* rs12917 polymorphism in cancer risk: an updated pooling analysis with 21010 cases and 34018 controls

**DOI:** 10.1042/BSR20180942

**Published:** 2018-10-15

**Authors:** Zhiguo Sheng, Meini Kang, Hao Wang

**Affiliations:** 1 Department of Neurosurgery, The First Center Hospital of Tianjin, Tianjin Medical University First Center Clinical College, Tianjin 300192, P.R. China; 2 Department of Family Medicine, Tianjin United Family Hospital, Tianjin 300221, P.R. China; 3 Department of Neurosurgery, Shenzhen People’s Hospital, The Second Clinical Medical College of Jinan University, Shenzhen 518020, P.R. China

**Keywords:** MGMT, polymorphism, Cancer, meta-analysis

## Abstract

In the present study, we aimed at determining the potential role of rs12917 polymorphism of the *O*-6-methylguanine-DNA methyltransferase (*MGMT*) gene in the occurrence of cancer. Based on the available data from the online database, we performed an updated meta-analysis. We retrieved 537 articles from our database research and finally selected a total of 54 case–control studies (21010 cases and 34018 controls) for a series of pooling analyses. We observed an enhanced risk in cancer cases compared with controls, using the genetic models T/T compared with C/C (*P*-value of association test <0.001; odds ratio (OR) = 1.29) and T/T compared with C/C+C/T (*P*<0.001; OR = 1.32). We detected similar positive results in the subgroups ‘Caucasian’, and ‘glioma’ (all *P*<0.05; OR > 1). However, we detected negative results in our analyses of most of the other subgroups (*P*>0.05). Begg’s and Egger’s tests indicated that the results were free of potential publication bias, and sensitivity analysis suggested the stability of the pooling results. In summary, the T/T *genotype of MGMT* rs12917 is likely to be linked to an enhanced susceptibility to cancer overall, especially glioma, in the Caucasian population.

## Introduction

In humans, the *O*-6-methylguanine-DNA methyltransferase (MGMT) protein, encoded by the *MGMT* gene located on chromosome 10 (10q26) [1], is involved in the DNA repair process [2,3]. By means of methyl transfer, MGMT removes alkylating agents from the DNA direct reversal repair pathway and thus repairs the DNA [2,3]. Two potential functional polymorphisms have been identified in the *MGMT* gene, namely rs12917 (Leu84Phe) and rs2308321 (Ile143Val) [4,5]. In addition, the promoter methylation status of the gene is reportedly correlated with several clinical diseases, such as glioblastoma [6,7], gastric cancer [8], and oral carcinoma [9].

Both genetic and environmental factors contribute to the occurrence and progression of clinical cancers [10,11]. A number of studies have been conducted on the potential genetic effect of *MGMT* rs12917 polymorphism on its susceptibility to cancer, but the results were inconclusive. Before 2013, only three relative meta-analyses investigated the potential role of this polymorphism in the overall risk for cancer [12–14]. Based on the currently available data, we performed an updated meta-analysis to reassess the genetic relationship between *MGMT* rs12917 polymorphism and cancer risk. We enrolled a total of 54 case–control studies for the study.

## Materials and methods

### Database searching strategy

To identify potential publications, we searched four online electronic databases (PubMed, Embase, Cochrane Library, and WANFANG) up through August 2018. We used the terms ‘MeSH (Medical Subject Headings)’ and ‘Entry Terms’ to search PubMed and Cochrane Library, and ‘Emtree’ and ‘Synonyms’ for Embase. The search string we used for PubMed was as follows: (((((((((((((((O(6)-Methylguanine-DNA Methyltransferase [MeSH Terms]) OR Methylated-DNA-Protein-Cysteine S-Methyltransferase) OR Methylated DNA Protein Cysteine S Methyltransferase) OR S-Methyltransferase, Methylated-DNA-Protein- Cysteine) OR O(6)-Methylguanine Methyltransferase) OR O(6)-Alkylguanine-DNA Alkyltransferase) OR O(6)-MeG-DNA Methyltransferase) OR O(6)-Methylguanine DNA Transmethylase) OR Guanine-O(6)-Alkyltransferase) OR O(6)-AGT) OR DNA Repair Methyltransferase II) OR DNA Repair Methyltransferase I) OR MGMT)) AND ((((((((Polymorphism, Genetic [MeSH Terms]) OR Polymorphisms, Genetic) OR Genetic Polymorphisms) OR Genetic Polymorphism) OR Polymorphism (Genetics)) OR Polymorphisms (Genetics)) OR Polymorphism) OR Polymorphisms)) AND ((((((((((((((((((Neoplasms [MeSH Terms]) OR Neoplasia) OR Neoplasias) OR Neoplasm) OR Tumors) OR Tumor) OR Cancer) OR Cancers) OR Malignant Neoplasms) OR Malignant Neoplasm) OR Neoplasm, Malignant) OR Neoplasms, Malignant) OR Malignancy) OR Malignancies) OR Benign Neoplasms) OR Neoplasms, Benign) OR Benign Neoplasm) OR Neoplasm, Benign).

### Article screening strategy

We designed our inclusion and exclusion criteria according to Patient, Intervention, Comparison and Outcome and Study design (PICOS) principles. We ruled out duplicates and screened improper articles. Exclusion criteria were as follows: (P), non-cancer patients; (I), other variants, gene expression or methylation; (C), lack of study controls or *P*-value of Hardy–Weinberg equilibrium (HWE) <0.05; (O), lack of full genotype frequency data; (S), review, meta, poster, or meeting abstract. Eligible articles had to be designed as case–control studies, targetting the genetic relationship between *MGMT* rs12917 and cancer risk and containing the full genotype (C/C, C/T, T/T) frequencies in both cancer cases and negative controls.

### Data extraction and quality assessment

After extracting usable data, we listed the basic information in tables. We assessed methodological quality via the Newcastle–Ottawa Scale (NOS) [15]. High-quality articles with NOS score > 5 were regarded as eligible and included in our statistical analysis.

### Statistical analysis

We used STATA software version 12.0-SE (StataCorp, College Station, TX) to perform our analyses. We first assessed the inter-study heterogeneity using Cochran’s Q statistic and the I^2^ test. A *P*-value of Cochran’s Q statistic < 0.1 or *I^2^* value > 50% was considered to show a high level of heterogeneity. We thus used the DerSimonian–Laird association test with a random-effects model. Otherwise, we used the Mantel–Haenszel association test with a fixed-effects model. The *P*-value of association test, summary odds ratio (OR), along with the corresponding 95% confidence interval (CI) could be obtained for the allele (T compared with C), homozygous (T/T compared with C/C), recessive (T/T compared with C/C+C/T), heterozygous (C/T compared with C/C), dominant (C/T+T/T compared with C/C), and carrier (T compared with C) models.

We performed subgroup analyses by race, cancer type, and control source. Additionally, we assessed possible publication bias by means of Begg’s and Egger’s tests and evaluated the robustness of the results through sensitivity analysis.

## Results

### Eligible case–control studies

**Figure 1:**
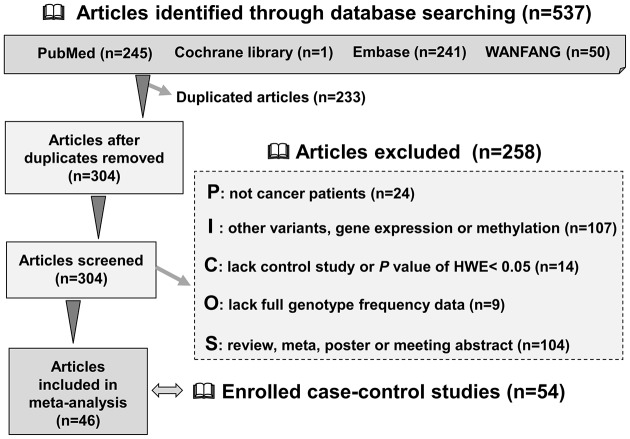
Flowchart for the identification of eligible case–control studies

Figure 1 depicts the flowchart for the identification of eligible case–control studies. We initially obtained a total of 537 articles by searching four databases, including PubMed (245 articles), Cochrane Library (1 article), Embase (241 articles), and WANFANG (50 articles). We then excluded 233 duplicates plus another 258 articles based strictly on our screening strategy. Finally, we identified 46 full-text articles for inclusion [4,5,16–59]. After data extraction and quality evaluation, we enrolled a total of 54 case–control studies free of poor quality (all NOS score > 5) in our pooling analyses. The basic information and genotype frequency distribution are presented in Supplementary Table S1 and Table 1, respectively.

**Table 1:** Genotype and allele frequency of *MGMT* rs12917 in the enrolled case–control studies

Authors	Year	Genotype (case)			Allele (case)		Cancer type (case)	Genotype (control)			Allele (control)		HWE (control)	
		C/C	C/T	T/T	C	T		C/C	C/T	T/T	C	T	*χ^2^*	*P*
**Agalliu et al.** [16]	**2010**	949	269	32	2167	333	Prostate cancer^**1**^	916	298	23	2130	344	0.05	0.83
		106	35	6	247	47	Prostate cancer^**2**^	60	20	1	140	22	0.22	0.64
**Akbari et al.** [17]	**2009**	142	53	1	337	55	Esophageal cancer	185	63	2	433	67	1.84	0.17
**Betti et al.** [18]	**2011**	95	36	2	226	40	MPM^**3**^	179	64	8	422	80	0.59	0.44
		50	17	1	117	19	MPM^**4**^	32	12	0	76	12	1.10	0.29
**Bye et al.** [19]	**2011**	225	111	10	561	131	Esophageal cancer^**1**^	300	155	14	755	183	1.28	0.26
		120	65	11	305	87	Esophageal cancer^**5**^	294	116	13	704	142	1.28	0.26
**Chae et al.** [20]	**2006**	344	84	4	772	92	Lung cancer	341	81	10	763	101	3.65	0.06
**Chuang et al.** [21]	**2011**	1105	307	43	2517	393	Head and neck cancer	2256	823	81	5335	985	0.33	0.57
**Doecke et al.** [22]	**2008**	416	136	14	968	164	Esophageal cancer	1029	281	27	2339	335	2.25	0.13
**Felini et al.** [23]	**2007**	289	84	6	662	96	Glioma	369	84	6	822	96	0.24	0.63
**Feng et al.** [24]	**2008**	96	58	47	250	152	Esophageal cancer	87	85	29	259	143	1.20	0.27
**Gu et al.** [25]	**2009**	152	60	2	364	64	Melanoma	168	43	1	379	45	1.01	0.31
**Hall et al.** [26]	**2007**	548	193	38	1289	269	UADT	730	281	23	1741	327	0.44	0.51
**Han et al.** [27]	**2006^1^**	344	82	8	770	98	Endometrial cancer	822	242	21	1886	284	0.42	0.52
**Han et al.** [28]	**2006^2^**	964	279	33	2207	345	Breast cancer	1,306	382	26	2994	434	0.10	0.75
**Hu et al.** [29]	**2013**	389	130	24	908	178	Glioma	405	84	6	894	96	0.48	0.49
**Hu et al.** [4]	**2007**	418	77	5	913	87	Lung cancer	421	93	3	935	99	0.78	0.38
**Huang et al.** [30]	**2017**	76	12	2	164	16	Glioma	75	14	1	164	16	0.14	0.71
**Huang et al.** [31]	**2007**	372	156	11	900	178	Cervical cancer	592	198	10	1382	218	2.12	0.15
**Huang et al.** [32]	**2010**	151	25	0	327	25	Oral cancer	89	21	0	199	21	1.22	0.27
**Huang et al.** [33]	**2005^1^**	190	82	8	462	98	Gastric cancer	279	99	9	657	117	0.00	0.95
**Huang et al.** [34]	**2005^2^**	386	117	11	889	139	Head and neck cancer	529	204	21	1262	246	0.06	0.80
**Inoue et al.** [35]	**2003**	55	18	0	128	18	Primary brain cancer	160	55	9	375	73	2.24	0.13
**Kiczmer** [36]	**2018**	49	11	9	109	29	Head and neck cancer	168	66	5	402	76	0.25	0.61
**Kietthubthew et al.** [37]	**2006**	84	21	1	189	23	Oral cancer	130	33	1	293	35	0.50	0.48
**Li et al.** [38]	**2005**	132	34	1	298	36	Bladder cancer	173	28	3	374	34	2.11	0.15
**Liu et al.** [39]	**2002^1^**	53	7	0	113	7	Lung cancer	89	11	0	189	11	0.34	0.56
**Liu et al.** [40]	**2002^2^**	21	3	0	45	3	Gynecologic tumor	89	11	0	189	11	0.34	0.56
		26	8	0	60	8	Digestive system cancer	89	11	0	189	11	0.34	0.56
**Liu et al.** [41]	**2006**	82	16	2	180	20	Esophageal cancer	57	8	0	122	8	0.28	0.60
**Liu et al.** [42]	**2009**	299	62	8	660	78	Glioma	267	89	7	623	103	0.02	0.89
**Loh et al.** [43]	**2011**	146	37	5	329	47	Cancer	894	212	14	2000	240	0.13	0.72
**Lu et al.** [44]	**2006**	142	45	4	329	53	Gastric cancer	186	59	6	431	71	0.26	0.61
**McKean-Cowdin et al.** [45]	**2009**	774	204	20	1752	244	Glioblastoma	1,480	453	35	3413	523	0.00	0.96
**O’Mara et al.** [46]	**2011**	889	261	23	2039	307	Endometrial cancer^**6**^	810	270	19	1890	308	0.42	0.52
		278	108	11	664	130	Endometrial cancer^**7**^	296	103	7	695	117	0.33	0.57
**Palli et al.** [47]	**2010**	210	77	4	497	85	Gastric cancer	395	131	11	921	153	0.00	0.97
**Rajaraman et al.** [48]	**2010**	265	77	9	607	95	Glioma	348	117	12	813	141	0.33	0.57
		102	23	4	227	31	Meningioma	348	117	12	813	141	0.33	0.57
		52	12	2	116	16	Acoustic neuroma	348	117	12	813	141	0.33	0.57
**Ritchey et al.** [49]	**2005**	123	36	2	282	40	Prostate cancer	213	32	1	458	34	0.03	0.86
**Shah et al.** [50]	**2012**	64	26	2	154	30	Esophageal cancer	57	20	0	134	20	1.72	0.19
**Shen et al.** [51]	**2005**	778	265	21	1821	307	Breast cancer	824	263	20	1911	303	0.03	0.85
**Shen et al.** [52]	**2007**	432	112	11	976	134	NHL	373	110	12	856	134	1.27	0.26
**Shi et al.** [53]	**2011**	253	47	3	553	53	AML	459	91	4	1009	99	0.05	0.83
**Stern et al.** [54]	**2007**	251	40	1	542	42	Colorectal cancer	959	194	13	2112	220	0.81	0.37
**Tranah et al.** [55]	**2006**	147	33	6	327	45	Colorectal cancer^**8**^	1,634	471	32	3739	535	0.09	0.77
		204	47	6	455	59	Colorectal cancer^**9**^	330	93	6	753	105	0.04	0.85
**Wang et al.** [5]	**2006**	832	259	30	1923	319	Lung cancer	872	272	19	2016	310	0.18	0.67
**Yang et al.** [56]	**2009**	33	14	1	80	16	NHL	289	58	5	636	68	1.10	0.29
**Zhang et al.** [57]	**2008**	352	53	1	757	55	Biliary track cancer	631	144	7	1406	158	0.15	0.70
**Zhang et al.** [58]	**2010**	563	151	7	1277	165	Head and neck cancer	933	284	17	2150	318	0.78	0.38
**Zienolddiny et al.** [59]	**2006**	189	102	13	480	128	Lung cancer	247	106	10	600	126	0.12	0.73

Abbreviations: AML, acute myeloid leukemia; MPM, malignant mesothelioma; NHL, non-Hodgkin’s lymphoma; UADT, upper aerodigestive tract.

**^1^**Data from Caucasian population. **^2^**Data from African population. **^3^**With population-based control. **^4^**With hospital-based control. **^5^**Data from mixed population. **^6^**Data from Australia. **^7^**Data from Poland. **^8^**With controls from Nurses’ Health Study (NHS). **^9^**With controls from Physicians’ Health Study (PHS) cohorts

### Meta-analysis data

**Figure 2:**
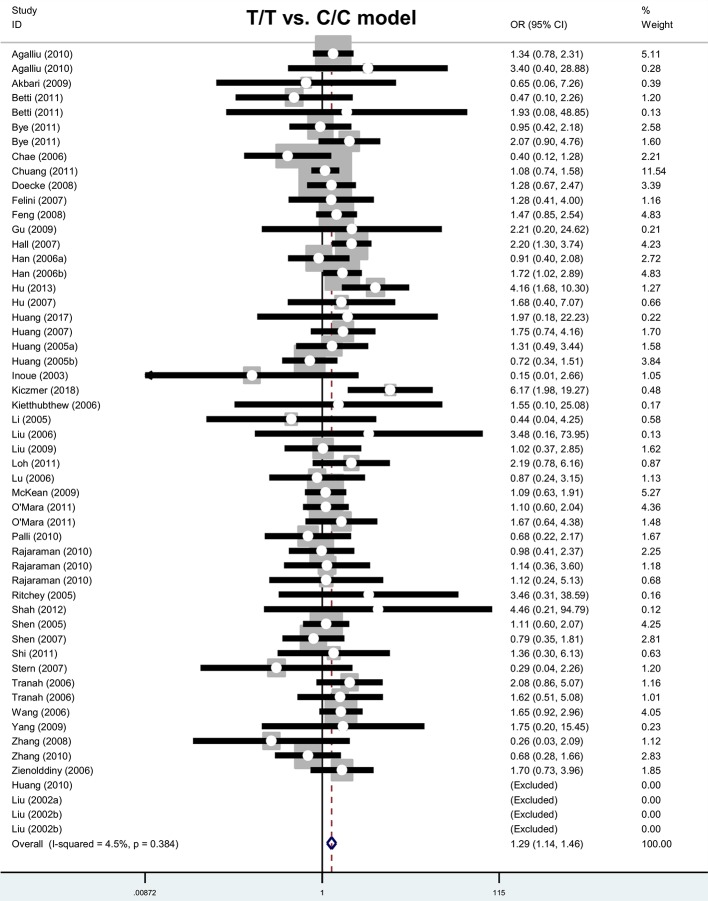
Forest plot of meta-analysis (T/T compared with C/C model)

First, we studied the association between the *MGMT* rs12917 polymorphism and cancer risk via an overall meta-analysis. As shown in Table 2, we included a total of 54 case–control studies with 21010 cases and 34018 controls under the genetic models of allele T compared with C, C/T compared with C/C, C/T+T/T compared with C/C, and carrier T compared with C; meanwhile, we included 50 studies with 20716 cases and 33608 controls under the models of T/T compared with C/C and T/T compared with C/C+C/T. For the homozygous, recessive and carrier genetic models, we performed a Mantel–Haenszel association test with a fixed-effects model, and we observed no high degree of heterogeneity (Table 2; all *P*-values of heterogeneity > 0.1; *I^2^* < 50%). For other models (all *P*-values of heterogeneity <0.001), we performed a DerSimonian–Laird association test with a random-effects model. Pooling data (Table 2) indicated an increased risk of cancer in cases compared with controls for the T/T compared with C/C (*P*-value of association test <0.001; OR = 1.29) and T/T compared with C/C+C/T (*P*<0.001; OR = 1.32) genetic models. Nevertheless, we failed to detect any statistical difference between cancer cases and negative controls under other genetic models (Table 2; all *P*>0.05). Forest plot data are shown in Figure 2 and Supplementary Figures S1–S5; they revealed that the T/T genotype of the *MGMT* rs12917 polymorphism was likely to be associated with an increased susceptibility to cancer.

**Table 2:** Meta-analysis of the association between *MGMT* rs12917 and cancer susceptibility

Models	Sample size			Heterogeneity		Association		
	Study	Case	Control	*I^2^*	*P*	Fixed/random	*P*	OR (95% CI)
**Allele T compared with C**	54	21010	34018	50.1%	<0.001	Random	0.354	-
**T/T compared with C/C**	50	20716	33608	4.5%	0.384	Fixed	<0.001	1.29 (1.14–1.46)
**T/T compared with C/C+C/T**	50	20716	33608	3.2%	0.410	Fixed	<0.001	1.32 (1.17–1.49)
**C/T compared with C/C**	54	21010	34018	46.1%	<0.001	Random	0.442	-
**C/T+T/T compared with C/C**	54	21010	34018	47.7%	<0.001	Random	0.976	-
**Carrier T compared with C**	54	21010	34018	20.0%	0.104	Fixed	0.642	-

-, OR (95% CI) data were not provided, when *P*-value of association >0.05.

### Subgroup analysis data

**Figure 3:**
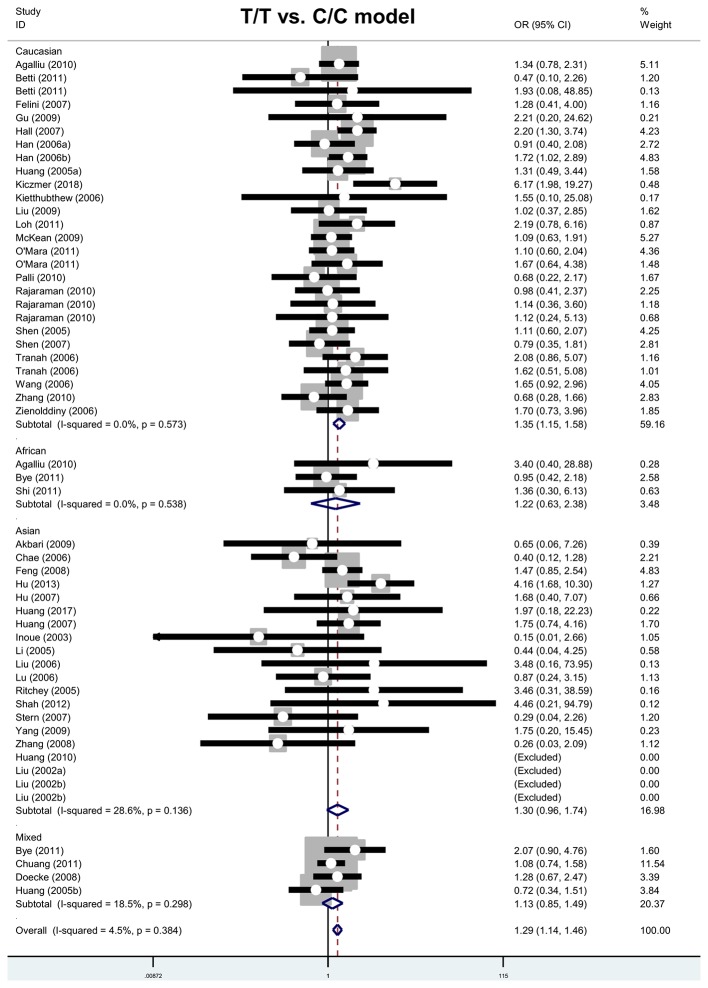
Forest plot of subgroup analysis by race (T/T compared with C/C model)

Next, we carried out four subgroup analyses by race, cancer type, and control source. For the T/T compared with C/C model (Table 3), the association test data showed an increased cancer risk in the subgroups ‘Caucasian’ (*P*<0.001; OR = 1.35), ‘glioma’ (*P*=0.022; OR = 1.70), ‘population-based control (PB)’ (*P*<0.001; OR = 1.32) and ‘hospital-based control (HB)’ (*P*<0.030; OR = 1.39). Figure 3 and Supplementary Figures S6–S7 present the forest plot data.

**Table 3:** Data of subgroup analysis under T/T compared with C/C model

Factor	Subgroup	Sample size			Heterogeneity		Association	
		Study	Case	Control	*I^2^*	*P*	*P*	OR (95% CI)
Race	Caucasian	27	13158	20678	0.0%	0.573	<0.001	1.35 (1.15, 1.58)
	African	3	796	1104	0.0%	0.538	0.560	-
	Asian	16	4031	6152	28.6%	0.136	0.088	-
Cancer type	Urinary system cancer	4	1725	1768	0.0%	0.526	0.174	-
	Esophageal cancer	8	2131	3907	0.0%	0.781	0.069	-
	Lung cancer	4	2357	2475	40.7%	0.167	0.155	-
	Head and neck cancer	14	5863	10581	39.5%	0.064	0.138	-
	Gastric cancer	3	762	1175	0.0%	0.692	0.891	-
	Blood cancer	3	906	1401	0.0%	0.702	0.882	-
	Colorectal cancer	3	735	3732	38.5%	0.197	0.416	-
	Brain cancer	9	2998	5030	17.4%	0.288	0.106	-
	Glioma	5	1735	1884	37.9%	0.168	0.022	1.70 (1.08, 2.68)
Control source	PB	39	16526	26488	6.3%	0.358	<0.001	1.32 (1.14, 1.52)
	HB	8	2482	4148	3.2%	0.405	0.030	1.39 (1.03, 1.86)

-, OR (95% CI) data were not provided, when *P-*value of association > 0.05.

For the T/T compared with C/C+C/T model (Table 4), we also observed positive correlations in the subgroups ‘Caucasian’ (*P*<0.001; OR = 1.37), ‘Asian’ (*P*=0.036; OR = 1.37), ‘glioma’ (*P*=0.026; OR = 1.68), ‘PB’ (*P*<0.001; OR = 1.32), and ‘HB’ (*P*=0.004; OR = 1.52). Supplementary Figures S8–S10 present the forest plot data.

**Table 4:** Data of subgroup analysis under T/T compared with C/C+C/T model

Factor	Subgroup	Sample size			Heterogeneity		Association	
		Study	Case	Control	I^2^	*P*	*P*	OR (95% CI)
Race	Caucasian	27	13158	20678	0.0%	0.528	<0.001	1.37 (1.17, 1.60)
	African	3	796	1104	0.0%	0.542	0.535	-
	Asian	16	4031	6152	27.2%	0.150	0.036	1.37 (1.02, 1.83)
Cancer type	Urinary system cancer	4	1725	1768	0.0%	0.527	0.152	-
	Esophageal cancer	8	2131	3907	0.0%	0.725	0.021	-
	Lung cancer	4	2357	2475	40.0%	0.467	0.174	-
	Head and neck cancer	14	5863	10581	37.5%	0.077	0.064	-
	Gastric cancer	3	762	1175	0.0%	0.718	0.815	-
	Blood cancer	3	906	1401	0.0%	0.769	0.901	-
	Colorectal cancer	3	735	3732	39.6%	0.191	0.344	-
	Brain cancer	9	2998	5030	3.0%	0.410	0.088	-
	Glioma	5	1735	1884	23.7%	0.263	0.026	1.68 (1.07, 2.65)
Control source	PB	39	16526	26488	2.5%	0.426	<0.001	1.32 (1.15, 1.52)
	HB	8	2482	4148	11.0%	0.344	0.004	1.52 (1.14, 2.03)

-, OR (95% CI) data was not provided, when *P*-value of association > 0.05.

We did not detect positive results for the other genetic models (Supplementary Tables S2–S5; *P*<0.05) except for the subgroups ‘colorectal cancer’ (Supplementary Table S3; *P*=0.041; OR = 0.79), ‘HB’ (Supplementary Table S3; *P*=0.027; OR = 0.86) under the C/T compared with C/C model; and the subgroup ‘head and neck cancer’ (Supplementary Table S5; *P*=0.020; OR = 0.92) under the carrier T compared with C model. Thus, the T/T genotype of *MGMT* rs12917 may have been associated with an increased risk of cancer in cases, especially the glioma cases, in the Caucasian population.

### Publication bias and sensitivity analysis

**Figure 4:**
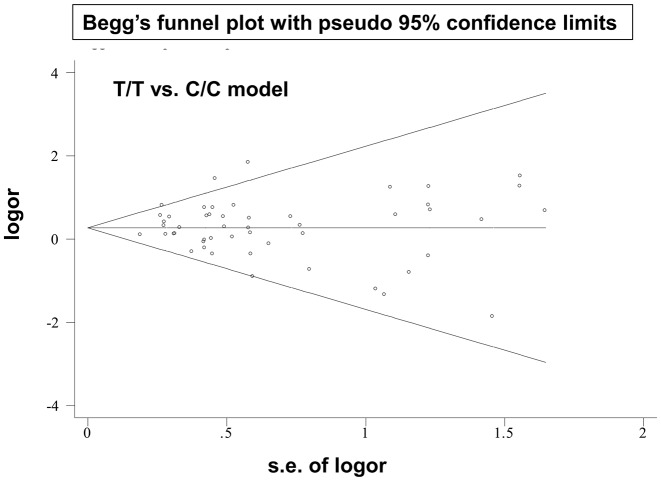
Begg’s funnel plot with pseudo-95% confidence limits (T/T compared with C/C model)

**Figure 5:**
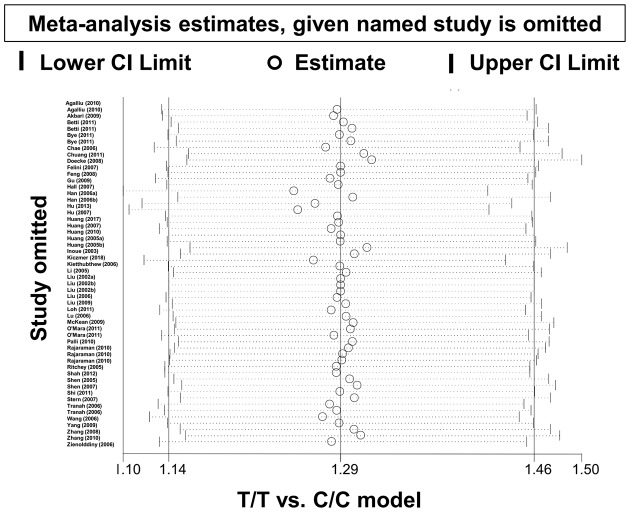
Sensitivity analysis result (T/T compared with C/C model)

Begg’s and Egger’s tests indicated that results were free of possible publication bias (Supplementary Table S6; *P*>0.05 for Begg’s test, >0.05 for Egger’s test). A Begg’s funnel plot with pseudo–95% confidence limits under the T/T compared with C/C model is shown in Figure 4. In addition, we observed the same stable results in our subsequent sensitivity analysis; data from this analysis under the homozygous model (Figure 5) are presented as an example.

## Discussion

We observed conflicting conclusions about the genetic role of *MGMT* rs12917 polymorphism in its susceptibility to different cancers. For instance, the polymorphism seems to be associated with the risk of esophageal cancer in the Chinese population [41], but not in the Kashmiri population [50]. This merits a quantitative synthesis via the meta-analytic approach. Although there were already three meta-analyses of the *MGMT* rs12917 polymorphism and its role in the overall risk for cancer [12–14], expanding the sample size and employing a distinct analysis strategy led to better results in our updated pooling analysis.

We did our best to gather candidate articles from four online databases. After screening them based on strict inclusion and exclusion criteria, we enrolled only the case–control studies that were of high quality and those that followed HWE. We ultimately included a total of 46 articles in our updated meta-analysis. After data extraction, we enrolled 54 case–control studies with 21010 cases and 34018 controls in the meta-analysis. We used the carrier, allele, homozygous, recessive, heterozygous, and dominant genetic models, and also confirmed the stability of the statistical results via sensitivity analysis.

In 2010, Zhong et al. [12] performed the first meta-analysis on this topic, reviewing 28 case–control studies from 26 articles [4,5,20,22,23,26–28,31,33–35,37,38,42,45,49,51,52,54,55,59–63]. Another 24 case–control studies [16–19,21,24,25,29,30,32,36,39–41,43,44,46–48,50,53,56–58] were included in our study. We excluded three studies not in-line with the HWE principle [61–63] and one that focussed only on colorectal adenomatous or hyperplastic polyps but not on colorectal cancer [60]. In 2013, Du et al. [14] enrolled 41 case–control studies with 16643 cancer cases and 26720 negative controls from 37 articles [5,16–20,22–24,26–28,31–34,37–41,43,44,46,47,49–59,64] in a meta-analysis. We excluded one of these studies [64] from our meta-analysis because it did not meet the requirement of full genotype frequency in both case and control groups. Finally, we enrolled another ten case–control studies [4,21,25,29,30,35,36,42,45,48]. In addition, when compared with another meta-analysis of Liu et al. (2013) [13], which consisted of 44 case–control studies from 37 articles [4,5,16,17,19,20,22,23,25–27,31–33,35,37,38,42,43,45–47,49,51,52,54–63,65,66], we excluded four studies that were not in HWE [61–63,66], one that did not analyze colorectal cancer [60], and one that included other genetic variants [65]. We also added another 15 new case–control studies [18,21,24,28–30,34,36,39–41,44,48,50,53] for the analysis.

Our updated pooling analysis data demonstrated that cases had an overall enhanced risk for cancer when compared with negative controls under the T/T compared with C/C and T/T compared with C/C+C/T genetic models, especially in the European-descended population, which is partly consistent with the data of previous analyses [12–14]. Moreover, we observed that the *MGMT* rs12917 polymorphism is likely to be associated with the susceptibility to glioma, which is partly in-line with the two studies on the association between DNA repair gene polymorphisms and glioma risk [67,68]. Nevertheless, owing to the limitation of sample size, the previous three meta-analyses of the overall risk for cancer did not conduct subgroup analyses of ‘glioma’ [12–14].

Some of the limitations to our meta-analysis are as follows:

1. Although the sample sizes enrolled were quite large (21010 cases and 34018 controls), genotype data were very limited in many subgroup analyses. For instance, we used only three case–control studies in our analyses of the subgroups for gastric [33,44,47], blood [52,53,56], and colorectal [54,55] cancers. Even for the subgroup analysis of ‘glioma’, with positive correlations under the T/T compared with C/C and T/T compared with C/C+C/T models, only five case–control studies [23,29,30,42,48] were included.
2. We did not investigate the genetic effects of the *MGMT* rs12917 polymorphism in combination with other variants, such as rs2308321 of *MGMT*, rs25487 of X-ray cross-complementing group 1 (*XRCC1*), and rs13181 of xeroderma pigmentosum complementation group D (*XPD*), in certain specific cancers.
3. We extracted certain demographic information such as the mean age at diagnosis and the sex of subject, but not other confounding factors such as lifestyle and clinical features. Moreover, we did not perform the relevant stratified meta-analyses due to lack of sufficient usable data.
4. We detected significant heterogeneity amongst studies under the allele T compared with C, C/T compared with C/C, C/T+T/T compared with C/C, and carrier T compared with C genetic models. Complicating factors such as race and cancer type may be sources of inter-study heterogeneity. For instance, we detected decreased levels of heterogeneity in the ‘Caucasian’ and ‘esophageal cancer’ subgroups. Although we observed a positive conclusion in the ‘glioma’ subgroup, we failed to detect reduced inter-study heterogeneity. Only five case–control studies [23,29,30,42,48] were enrolled.
5. There may be other undetected or unpublished articles containing potential eligible case–controls in other geographical locations or languages; in other words, our study may suffer from selection bias.
6. Last but most important, our meta-analysis found a positive conclusion between *MGMT* rs12917 and the risk of cancer in general for the T/T compared with C/C and T/T compared with C/C+C/T models. Considering the distinct etiopathogenesis or pathogenesis of different kinds of cancers, more studies of large-scale populations of different ethnicities are required for a more scientific elucidation of *MGMT* rs12917’s functional role in each particular cancer type.

To sum up, our updated pooling analysis offered additional evidence that *MGMT* rs12917 polymorphism is likely to be associated with an enhanced susceptibility to cancer overall, especially glioma, in the Caucasian population.

## Supporting information

**Figure S1:** 

**Figure S2:** 

**Figure S3:** 

**Figure S4:** 

**Figure S5:** 

**Figure S6:** 

**Figure S4:** 

**Figure S8:** 

**Figure S9:** 

**Figure S10:** 

**Table S1:** Basic information of included studies

**Table S2:** Data of subgroup analysis under allele T vs. C model

**Table S3:** Data of subgroup analysis under C/T vs. C/C model

**Table S4:** Data of subgroup analysis under C/T+T/T vs. C/C model

**Table S5:** Data of subgroup analysis under carrier T vs. C model

**Table S6:** Publication bias result

## Abbreviations

HB: hospital-based control
HWE: Hardy–Weinberg equilibrium
MeSH: Medical Subject Heading
*MGMT*: *O*-6-methylguanine-DNA methyltransferase
NOS: Newcastle–Ottawa scale
OR: odds ratio
PB: population-based control

## References

[1] Natarajan A.T., Vermeulen S., Darroudi F., Valentine M.B., Brent T.P., Mitra S. (1992) Chromosomal localization of human O6-methylguanine-DNA methyltransferase (MGMT) gene by in situ hybridization. Mutagenesis 7, 83–85 10.1093/mutage/7.1.83 1635460

[2] Christmann M., Verbeek B., Roos W.P. and Kaina B. (2011) O(6)-Methylguanine-DNA methyltransferase (MGMT) in normal tissues and tumors: enzyme activity, promoter methylation and immunohistochemistry. Biochim. Biophys. Acta 1816, 179–190 21745538 10.1016/j.bbcan.2011.06.002

[3] Nikolova T., Roos W.P., Kramer O.H., Strik H.M. and Kaina B. (2017) Chloroethylating nitrosoureas in cancer therapy: DNA damage, repair and cell death signaling. Biochim. Biophys. Acta 1868, 29–39 10.1016/j.bbcan.2017.01.004 28143714

[4] Hu Z., Wang H., Shao M., Jin G., Sun W., Wang Y. (2007) Genetic variants in MGMT and risk of lung cancer in Southeastern Chinese: a haplotype-based analysis. Hum. Mutat. 28, 431–440 10.1002/humu.20462 17285603

[5] Wang L., Liu H., Zhang Z., Spitz M.R. and Wei Q. (2006) Association of genetic variants of O6-methylguanine-DNA methyltransferase with risk of lung cancer in non-Hispanic Whites. Cancer Epidemiol. Biomarkers Prev. 15, 2364–2369 10.1158/1055-9965.EPI-06-0437 17164358

[6] Staedtke V., a Dzaye O.D. and Holdhoff M. (2016) Actionable molecular biomarkers in primary brain tumors. Trends Cancer 2, 338–349 10.1016/j.trecan.2016.06.003 28603776 PMC5461965

[7] Binabaj M.M., Bahrami A., ShahidSales S., Joodi M., Joudi Mashhad M. and Hassanian S.M. (2018) The prognostic value of MGMT promoter methylation in glioblastoma: a meta-analysis of clinical trials, J. Cell. Physiol. 233, 378–386, 10.1002/jcp.25896 28266716

[8] Zhang Z., Xin S., Gao M. and Cai Y. (2017) Promoter hypermethylation of MGMT gene may contribute to the pathogenesis of gastric cancer: A PRISMA-compliant meta-analysis. Medicine (Baltimore) 96, e6708 10.1097/MD.0000000000006708 28445279 PMC5413244

[9] Jayaprakash C., Radhakrishnan R., Ray S. and Satyamoorthy K. (2017) Promoter methylation of MGMT in oral carcinoma: A population-based study and meta-analysis. Arch. Oral Biol. 80, 197–208 10.1016/j.archoralbio.2017.04.006 28458179

[10] Theodoratou E., Timofeeva M., Li X., Meng X. and Ioannidis J.P.A. (2017) Nature, nurture, and cancer risks: genetic and nutritional contributions to cancer. Annu. Rev. Nutr. 37, 293–320 10.1146/annurev-nutr-071715-051004 28826375 PMC6143166

[11] Sugimura H. (2016) Susceptibility to human cancer: from the perspective of a pathologist. Pathol. Int. 66, 359–368 10.1111/pin.12418 27216305

[12] Zhong Y., Huang Y., Huang Y., Zhang T., Ma C., Zhang S. (2010) Effects of O6-methylguanine-DNA methyltransferase (MGMT) polymorphisms on cancer: a meta-analysis. Mutagenesis 25, 83–95 10.1093/mutage/gep050 19892775

[13] Liu J., Zhang R., Chen F., Yu C., Sun Y., Jia C. (2013) MGMT Leu84Phe polymorphism contributes to cancer susceptibility: evidence from 44 case-control studies. PLoS ONE 8, e75367 10.1371/journal.pone.0075367 24086516 PMC3784571

[14] Du L., Wang H., Xiong T., Ma Y., Yang J., Huang J. (2013) The polymorphisms in the MGMT gene and the risk of cancer: a meta-analysis. Tumour Biol. 34, 3227–3237 10.1007/s13277-013-0893-x 23760981

[15] Stang A. (2010) Critical evaluation of the Newcastle-Ottawa scale for the assessment of the quality of nonrandomized studies in meta-analyses. Eur. J. Epidemiol. 25, 603–605 10.1007/s10654-010-9491-z 20652370

[16] Agalliu I., Kwon E.M., Salinas C.A., Koopmeiners J.S., Ostrander E.A. and Stanford J.L. (2010) Genetic variation in DNA repair genes and prostate cancer risk: results from a population-based study. Cancer Causes Control 21, 289–300 10.1007/s10552-009-9461-5 19902366 PMC2811225

[17] Akbari M.R., Malekzadeh R., Shakeri R., Nasrollahzadeh D., Foumani M., Sun Y. (2009) Candidate gene association study of esophageal squamous cell carcinoma in a high-risk region in Iran. Cancer Res. 69, 7994–8000 10.1158/0008-5472.CAN-09-1149 19826048 PMC3505030

[18] Betti M., Ferrante D., Padoan M., Guarrera S., Giordano M., Aspesi A. (2011) XRCC1 and ERCC1 variants modify malignant mesothelioma risk: a case-control study. Mutat. Res. 708, 11–20 10.1016/j.mrfmmm.2011.01.001 21277872

[19] Bye H., Prescott N.J., Matejcic M., Rose E., Lewis C.M., Parker M.I. (2011) Population-specific genetic associations with oesophageal squamous cell carcinoma in South Africa. Carcinogenesis 32, 1855–1861 10.1093/carcin/bgr211 21926110 PMC3220606

[20] Chae M.H., Jang J.S., Kang H.G., Park J.H., Park J.M., Lee W.K. (2006) O6-alkylguanine-DNA alkyltransferase gene polymorphisms and the risk of primary lung cancer. Mol. Carcinog. 45, 239–249 10.1002/mc.20171 16385589

[21] Chuang S.C., Agudo A., Ahrens W., Anantharaman D., Benhamou S., Boccia S. (2011) Sequence variants and the risk of head and neck cancer: pooled analysis in the INHANCE consortium. Front. Oncol. 1, 13 10.3389/fonc.2011.00013 22655231 PMC3356135

[22] Doecke J., Zhao Z.Z., Pandeya N., Sadeghi S., Stark M., Green A.C. (2008) Polymorphisms in MGMT and DNA repair genes and the risk of esophageal adenocarcinoma. Int. J. Cancer 123, 174–180 10.1002/ijc.23410 18386788

[23] Felini M.J., Olshan A.F., Schroeder J.C., North K.E., Carozza S.E., Kelsey K.T. (2007) DNA repair polymorphisms XRCC1 and MGMT and risk of adult gliomas. Neuroepidemiology 29, 55–58 10.1159/000108919 17898525

[24] Feng X.X., Li Z.F., Wang L.B., Zhang J.B. and Lu Z.X. (2008) Relationship between MGMT gene polymorphism and susceptibility of esophageal cancer. Zhong Guo Gong Gong Wei Sheng 24, 697–699

[25] Gu F., Qureshi A.A., Kraft P., Guo Q., Hunter D.J. and Han J. (2009) Polymorphisms in genes involved in DNA repair, cell growth, oxidative stress and inflammatory response, and melanoma risk. Br. J. Dermatol. 161, 209–212 10.1111/j.1365-2133.2009.09219.x 19438866 PMC2709292

[26] Hall J., Hashibe M., Boffetta P., Gaborieau V., Moullan N., Chabrier A. (2007) The association of sequence variants in DNA repair and cell cycle genes with cancers of the upper aerodigestive tract. Carcinogenesis 28, 665–671 10.1093/carcin/bgl160 17040931

[27] Han J., Hankinson S.E. and De Vivo I. (2006) Polymorphisms in O6-methylguanine DNA methyltransferase and endometrial cancer risk. Carcinogenesis 27, 2281–2285 10.1093/carcin/bgl099 16777993

[28] Han J., Tranah G.J., Hankinson S.E., Samson L.D. and Hunter D.J. (2006) Polymorphisms in O6-methylguanine DNA methyltransferase and breast cancer risk. Pharmacogenet. Genomics 16, 469–474 10.1097/01.fpc.0000215065.21718.4c 16788379

[29] Hu Y.Z., Wang D.H., Gong H.D., Li G.Z., Li J.W., Wang L.J. (2013) Correlation of polymorphism of DNA repair gene XRCC1 and MGMT with susceptibility to glioma in a Han population in northeastern China. Chin. J. Cancer Prev. Treat. 20, 1629–1633

[30] Huang G.Y., Zhang X., Wu Y.J., Cao C.N., Pu J., Ming Y. (2017) Association between genetic polymorphisms of MGMT and CASP3 and the susceptibility to glioma. Guizhou Med. J. 41, 118–140

[31] Huang J., Ye F., Chen H., Lu W. and Xie X. (2007) Amino acid substitution polymorphisms of the DNA repair gene MGMT and the susceptibility to cervical carcinoma. Carcinogenesis 28, 1314–1322 10.1093/carcin/bgm003 17234722

[32] Huang S.H., Chang P.Y., Liu C.J., Lin M.W. and Hsia K.T. (2010) O6-methylguanine-DNA methyltransferase gene coding region polymorphisms and oral cancer risk. J. Oral Pathol. Med. 39, 645–650 10.1111/j.1600-0714.2009.00880.x 20412404

[33] Huang W.Y., Chow W.H., Rothman N., Lissowska J., Llaca V., Yeager M. (2005) Selected DNA repair polymorphisms and gastric cancer in Poland. Carcinogenesis 26, 1354–1359 10.1093/carcin/bgi084 15802298

[34] Huang W.Y., Olshan A.F., Schwartz S.M., Berndt S.I., Chen C., Llaca V. (2005) Selected genetic polymorphisms in MGMT, XRCC1, XPD, and XRCC3 and risk of head and neck cancer: a pooled analysis. Cancer Epidemiol Biomarkers Prev. 14, 1747–1753 10.1158/1055-9965.EPI-05-0162 16030112

[35] Inoue R., Isono M., Abe M., Abe T. and Kobayashi H. (2003) A genotype of the polymorphic DNA repair gene MGMT is associated with *de novo* glioblastoma. Neurol. Res 25, 875–879 10.1179/016164103771954005 14669534

[36] Kiczmer P., Prawdzic Senkowska A., Strzelczyk J.K., Szydlo B., Biernacki K., Osadnik T. (2018) The role of MGMT polymorphisms rs12917 and rs11016879 in head and neck cancer risk and prognosis. Acta Biochim. Pol. 65, 87–92 10.18388/abp.2017_1613 29370316

[37] Kietthubthew S., Sriplung H., Au W.W. and Ishida T. (2006) Polymorphism in DNA repair genes and oral squamous cell carcinoma in Thailand. Int. J. Hyg. Environ. Health 209, 21–29 10.1016/j.ijheh.2005.06.002 16373199

[38] Li C., Liu J., Li A., Qian L., Wang X., Wei Q. (2005) Exon 3 polymorphisms and haplotypes of O6-methylguanine-DNA methyltransferase and risk of bladder cancer in southern China: a case-control analysis. Cancer Lett. 227, 49–57 10.1016/j.canlet.2005.03.043 15885889

[39] Liu R.Q. and Zhuang Z.X. (2002) Single-nucleotide polymorphisms of human O6-methylguanine-DNA methyltransferase (MGMT) gene in lung cancer patients from south china. Wei Sheng Du Li Xue Za Zhi 16, 1–5

[40] Liu R.Q., Zhuang Z.X., He C.H. and He Y. (2002) Relationship between genetic polymorphisms of human O6-methylguanine-DNA methyltransferase (MGMT) gene. Ai Zheng Ji Bian Tu Bian 14, 101–106

[41] Liu S.H., Su M., Cheng L., Sun B.L. and Lu Z.H. (2006) Polymorphisms of O6-methylguanine-DNA methyltransferase gene in Chinese Chaoshan esophageal cancer patients. Ai Zheng Ji Bian Tu Bian 18, 100–104

[42] Liu Y., Scheurer M.E., El-Zein R., Cao Y., Do K.A., Gilbert M. (2009) Association and interactions between DNA repair gene polymorphisms and adult glioma. Cancer Epidemiol. Biomarkers Prev. 18, 204–214 10.1158/1055-9965.EPI-08-0632 19124499 PMC2917049

[43] Loh Y.H., Mitrou P.N., Wood A., Luben R.N., McTaggart A., Khaw K.T. (2011) SMAD7 and MGMT genotype variants and cancer incidence in the European Prospective Investigation into Cancer and Nutrition (EPIC)-Norfolk Study. Cancer Epidemiol. 35, 369–374 10.1016/j.canep.2010.09.011 21075068

[44] Lu Y., Xu Y.C., Shen J., Yu R.B., Niu J.Y. and Guo J.T. (2006) Study on the association between the role of polymorphisms of the O6-methylguanine-DNA methyltransferase gene and gastric cancer hereditary susceptibility. Ji Bing Kong Zhi Za Zhi 10, 222–225

[45] McKean-Cowdin R., Barnholtz-Sloan J., Inskip P.D., Ruder A.M., Butler M., Rajaraman P. (2009) Associations between polymorphisms in DNA repair genes and glioblastoma. Cancer Epidemiol. Biomarkers Prev. 18, 1118–1126 10.1158/1055-9965.EPI-08-1078 19318434 PMC2667563

[46] O’Mara T.A., Ferguson K., Fahey P., Marquart L., Yang H.P., Lissowska J. (2011) CHEK2, MGMT, SULT1E1 and SULT1A1 polymorphisms and endometrial cancer risk. Twin Res. Hum. Genet. 14, 328–332 10.1375/twin.14.4.328 21787115 PMC4119964

[47] Palli D., Polidoro S., D’Errico M., Saieva C., Guarrera S., Calcagnile A.S. (2010) Polymorphic DNA repair and metabolic genes: a multigenic study on gastric cancer. Mutagenesis 25, 569–575 10.1093/mutage/geq042 20817763

[48] Rajaraman P., Hutchinson A., Wichner S., Black P.M., Fine H.A., Loeffler J.S. (2010) DNA repair gene polymorphisms and risk of adult meningioma, glioma, and acoustic neuroma. Neuro Oncol. 12, 37–48 10.1093/neuonc/nop012 20150366 PMC2940551

[49] Ritchey J.D., Huang W.Y., Chokkalingam A.P., Gao Y.T., Deng J., Levine P. (2005) Genetic variants of DNA repair genes and prostate cancer: a population-based study. Cancer Epidemiol. Biomarkers Prev. 14, 1703–1709 10.1158/1055-9965.EPI-04-0809 16030105

[50] Shah M.A., Shaff S.M., Lone G.N. and Jan S.M. (2012) Lack of influence of MGMT codon Leu84Phe and codon Ileu143Val polymorphisms on esophageal cancer risk in the Kashmir valley. Asian Pac. J. Cancer Prev. 13, 3047–3052 10.7314/APJCP.2012.13.7.3047 22994708

[51] Shen J., Terry M.B., Gammon M.D., Gaudet M.M., Teitelbaum S.L., Eng S.M. (2005) MGMT genotype modulates the associations between cigarette smoking, dietary antioxidants and breast cancer risk. Carcinogenesis 26, 2131–2137 10.1093/carcin/bgi179 16014702

[52] Shen M., Purdue M.P., Kricker A., Lan Q., Grulich A.E., Vajdic C.M. (2007) Polymorphisms in DNA repair genes and risk of non-Hodgkin’s lymphoma in New South Wales, Australia. Haematologica 92, 1180–1185 10.3324/haematol.11324 17666372

[53] Shi J.Y., Ren Z.H., Jiao B., Xiao R., Yun H.Y., Chen B. (2011) Genetic variations of DNA repair genes and their prognostic significance in patients with acute myeloid leukemia. Int. J. Cancer 128, 233–238 10.1002/ijc.25318 20232390

[54] Stern M.C., Conti D.V., Siegmund K.D., Corral R., Yuan J.M., Koh W.P. (2007) DNA repair single-nucleotide polymorphisms in colorectal cancer and their role as modifiers of the effect of cigarette smoking and alcohol in the Singapore Chinese Health Study. Cancer Epidemiol. Biomarkers Prev. 16, 2363–2372 10.1158/1055-9965.EPI-07-0268 18006925

[55] Tranah G.J., Bugni J., Giovannucci E., Ma J., Fuchs C., Hines L. (2006) O6-methylguanine-DNA methyltransferase Leu84Phe and Ile143Val polymorphisms and risk of colorectal cancer in the Nurses’ Health Study and Physicians’ Health Study (United States). Cancer Causes Control 17, 721–731 10.1007/s10552-006-0005-y 16633920

[56] Yang F., Shi J.Y., Xu L., Ren L.J., Zhang Q.H., Zhao W.L. (2009) Genetic susceptibility of single nucleotide polymorphism in MGMT to non-Hodgkin lymphoma. Zhonghua Xue Ye Xue Za Zhi 30, 622–625 19954624

[57] Zhang M., Huang W.Y., Andreotti G., Gao Y.T., Rashid A., Chen J. (2008) Variants of DNA repair genes and the risk of biliary tract cancers and stones: a population-based study in China. Cancer Epidemiol. Biomarkers Prev. 17, 2123–2127 10.1158/1055-9965.EPI-07-2735 18708406 PMC2860746

[58] Zhang Z., Wang L., Wei S., Liu Z., Wang L.E., Sturgis E.M. (2010) Polymorphisms of the DNA repair gene MGMT and risk and progression of head and neck cancer. DNA Repair (Amst.) 9, 558–566 10.1016/j.dnarep.2010.02.006 20206583 PMC2883263

[59] Zienolddiny S., Campa D., Lind H., Ryberg D., Skaug V., Stangeland L. (2006) Polymorphisms of DNA repair genes and risk of non-small cell lung cancer. Carcinogenesis 27, 560–567 10.1093/carcin/bgi232 16195237

[60] Bigler J., Ulrich C.M., Kawashima T., Whitton J. and Potter J.D. (2005) DNA repair polymorphisms and risk of colorectal adenomatous or hyperplastic polyps. Cancer Epidemiol. Biomarkers Prev. 14, 2501–2508 10.1158/1055-9965.EPI-05-0270 16284370

[61] Jiao L., Bondy M.L., Hassan M.M., Wolff R.A., Evans D.B., Abbruzzese J.L. (2006) Selected polymorphisms of DNA repair genes and risk of pancreatic cancer. Cancer Detect. Prev. 30, 284–291 10.1016/j.cdp.2006.05.002 16844323 PMC1857309

[62] Krzesniak M., Butkiewicz D., Samojedny A., Chorazy M. and Rusin M. (2004) Polymorphisms in TDG and MGMT genes - epidemiological and functional study in lung cancer patients from Poland. Ann. Hum. Genet. 68, 300–312 10.1046/j.1529-8817.2004.00079.x 15225156

[63] Moreno V., Gemignani F., Landi S., Gioia-Patricola L., Chabrier A., Blanco I. (2006) Polymorphisms in genes of nucleotide and base excision repair: risk and prognosis of colorectal cancer. Clin. Cancer Res. 12, 2101–2108 10.1158/1078-0432.CCR-05-1363 16609022

[64] Hung R.J., Baragatti M., Thomas D., McKay J., Szeszenia-Dabrowska N., Zaridze D. (2007) Inherited predisposition of lung cancer: a hierarchical modeling approach to DNA repair and cell cycle control pathways. Cancer Epidemiol. Biomarkers Prev. 16, 2736–2744 10.1158/1055-9965.EPI-07-0494 18086781

[65] Hazra A., Chanock S., Giovannucci E., Cox D.G., Niu T., Fuchs C. (2008) Large-scale evaluation of genetic variants in candidate genes for colorectal cancer risk in the nurses’ health study and the health professionals’ follow-up study. Cancer Epidemiol. Biomarkers Prev. 17, 311–319 10.1158/1055-9965.EPI-07-0195 18268114

[66] Khatami F., Noorinayer B., Mohebi S.R., Ghiasi S., Mohebi R., Hashemi M. (2009) Effects of amino acid substitution polymorphisms of two DNA methyltransferases on susceptibility to sporadic colorectal cancer. Asian Pac. J. Cancer Prev. 10, 1183–1188 20192566

[67] Adel Fahmideh M., Schwartzbaum J., Frumento P. and Feychting M. (2014) Association between DNA repair gene polymorphisms and risk of glioma: a systematic review and meta-analysis. Neuro Oncol. 16, 807–814 10.1093/neuonc/nou003 24500421 PMC4022225

[68] Liu K. and Jiang Y. (2017) Polymorphisms in DNA repair gene and susceptibility to glioma: a systematic review and meta-analysis based on 33 studies with 15 SNPs in 9 genes. Cell Mol. Neurobiol. 37, 263–274 10.1007/s10571-016-0367-y 27055523 PMC11482202

