# Correction: The potential role of MGMT rs12917 polymorphism in cancer risk: an updated pooling analysis with 21010 cases and 34018 controls

**DOI:** 10.1042/BSR-20180942_COR

**Published:** 2019-01-11

**Authors:** Zhiguo Sheng, Meini Kang, Hao Wang

***Bioscience Reports* (2018) 38, BSR20180942; https://doi.org/10.1042/BSR20180942**

The following source of funding was not acknowledged in the published version of the article:

This work was supported by grants from the key program of Tianjin Health Bureau (Grant NO.14KG104).

The online version was corrected on 20 December 2018.

