# Supplementary material for: The potential role of MGMT rs12917 polymorphism in cancer risk: an updated pooling analysis with 21010 cases and 34018 controls
Source: Biosci Rep. 2018 Oct 15;38(5):BSR20180942. doi: 10.1042/BSR20180942 (PMC6435461; doi:10.1042/BSR20180942)
Supplement: Supplementary file 1 [file bsr20180942_Supp1.pdf]

Figure S1

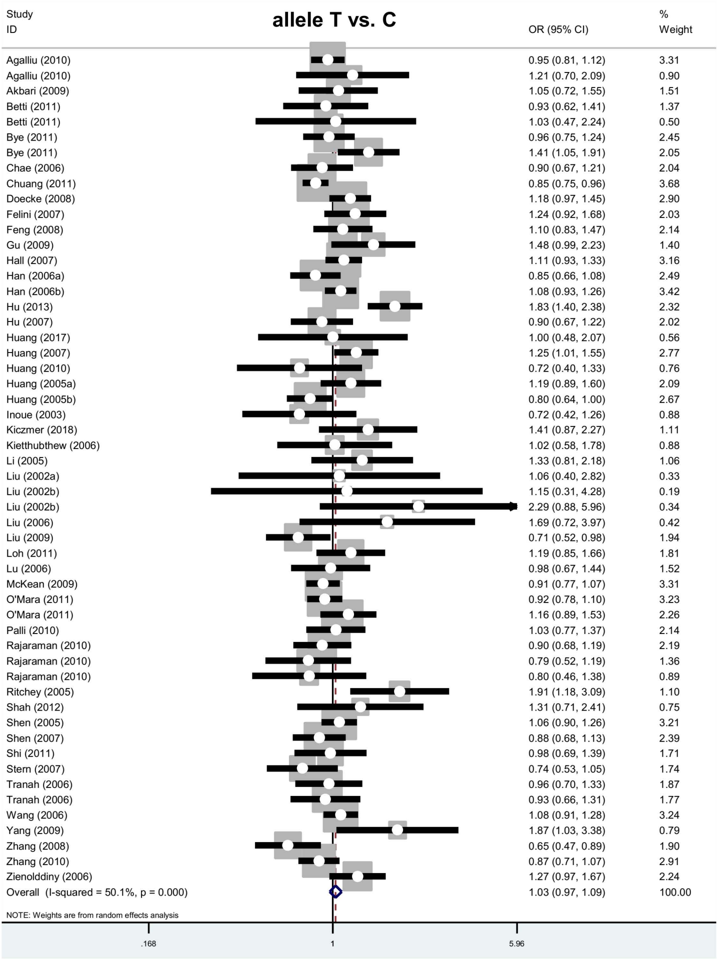

Study
ID
allele T vs. C
OR (95% CI)
%
Weight
Agalliu (2010) 0.95 (0.81, 1.12) 3.31
Agalliu (2010) 1.21 (0.70, 2.09) 0.90
Akbari (2009) 1.05 (0.72, 1.55) 1.51
Betti (2011) 0.93 (0.62, 1.41) 1.37
Betti (2011) 1.03 (0.47, 2.24) 0.50
Bye (2011) 0.96 (0.75, 1.24) 2.45
Bye (2011) 1.41 (1.05, 1.91) 2.05
Chae (2006) 0.90 (0.67, 1.21) 2.04
Chuang (2011) 0.85 (0.75, 0.96) 3.68
Doecke (2008) 1.18 (0.97, 1.45) 2.90
Felini (2007) 1.24 (0.92, 1.68) 2.03
Feng (2008) 1.10 (0.83, 1.47) 2.14
Gu (2009) 1.48 (0.99, 2.23) 1.40
Hall (2007) 1.11 (0.93, 1.33) 3.16
Han (2006a) 0.85 (0.66, 1.08) 2.49
Han (2006b) 1.08 (0.93, 1.26) 3.42
Hu (2013) 1.83 (1.40, 2.38) 2.32
Hu (2007) 0.90 (0.67, 1.22) 2.02
Huang (2017) 1.00 (0.48, 2.07) 0.56
Huang (2007) 1.25 (1.01, 1.55) 2.77
Huang (2010) 0.72 (0.40, 1.33) 0.76
Huang (2005a) 1.19 (0.89, 1.60) 2.09
Huang (2005b) 0.80 (0.64, 1.00) 2.67
Inoue (2003) 0.72 (0.42, 1.26) 0.88
Kiczmer (2018) 1.41 (0.87, 2.27) 1.11
Kietthubthew (2006) 1.02 (0.58, 1.78) 0.88
Li (2005) 1.33 (0.81, 2.18) 1.06
Liu (2002a) 1.06 (0.40, 2.82) 0.33
Liu (2002b) 1.15 (0.31, 4.28) 0.19
Liu (2002b) 2.29 (0.88, 5.96) 0.34
Liu (2006) 1.69 (0.72, 3.97) 0.42
Liu (2009) 0.71 (0.52, 0.98) 1.94
Loh (2011) 1.19 (0.85, 1.66) 1.81
Lu (2006) 0.98 (0.67, 1.44) 1.52
McKean (2009) 0.91 (0.77, 1.07) 3.31
O'Mara (2011) 0.92 (0.78, 1.10) 3.23
O'Mara (2011) 1.16 (0.89, 1.53) 2.26
Palli (2010) 1.03 (0.77, 1.37) 2.14
Rajaraman (2010) 0.90 (0.68, 1.19) 2.19
Rajaraman (2010) 0.79 (0.52, 1.19) 1.36
Rajaraman (2010) 0.80 (0.46, 1.38) 0.89
Ritchey (2005) 1.91 (1.18, 3.09) 1.10
Shah (2012) 1.31 (0.71, 2.41) 0.75
Shen (2005) 1.06 (0.90, 1.26) 3.21
Shen (2007) 0.88 (0.68, 1.13) 2.39
Shi (2011) 0.98 (0.69, 1.39) 1.71
Stern (2007) 0.74 (0.53, 1.05) 1.74
Tranah (2006) 0.96 (0.70, 1.33) 1.87
Tranah (2006) 0.93 (0.66, 1.31) 1.77
Wang (2006) 1.08 (0.91, 1.28) 3.24
Yang (2009) 1.87 (1.03, 3.38) 0.79
Zhang (2008) 0.65 (0.47, 0.89) 1.90
Zhang (2010) 0.87 (0.71, 1.07) 2.91
Zienolddiny (2006) 1.27 (0.97, 1.67) 2.24
Overall (I-squared = 50.1%, p = 0.000) 1.03 (0.97, 1.09) 100.00
NOTE: Weights are from random effects analysis
.168
1
5.96

Figure S2

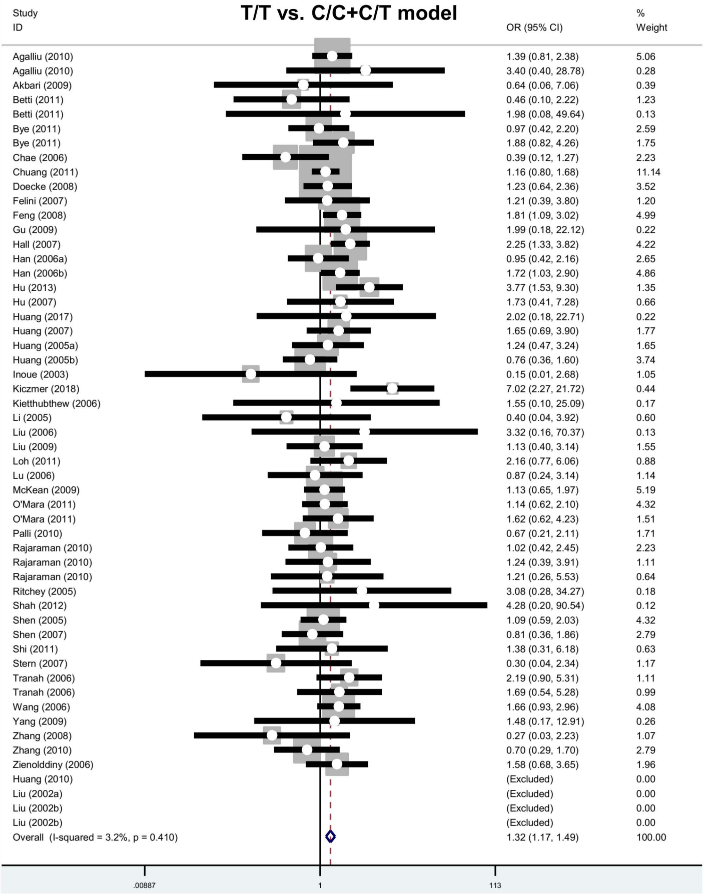
T/T vs. C/C+C/T model
Study ID
OR (95% CI)
% Weight
Agalliu (2010) 1.39 (0.81, 2.38) 5.06
Agalliu (2010) 3.40 (0.40, 28.78) 0.28
Akbari (2009) 0.64 (0.06, 7.06) 0.39
Betti (2011) 0.46 (0.10, 2.22) 1.23
Betti (2011) 1.98 (0.08, 49.64) 0.13
Bye (2011) 0.97 (0.42, 2.20) 2.59
Bye (2011) 1.88 (0.82, 4.26) 1.75
Chae (2006) 0.39 (0.12, 1.27) 2.23
Chuang (2011) 1.16 (0.80, 1.68) 11.14
Doecke (2008) 1.23 (0.64, 2.36) 3.52
Felini (2007) 1.21 (0.39, 3.80) 1.20
Feng (2008) 1.81 (1.09, 3.02) 4.99
Gu (2009) 1.99 (0.18, 22.12) 0.22
Hall (2007) 2.25 (1.33, 3.82) 4.22
Han (2006a) 0.95 (0.42, 2.16) 2.65
Han (2006b) 1.72 (1.03, 2.90) 4.86
Hu (2013) 3.77 (1.53, 9.30) 1.35
Hu (2007) 1.73 (0.41, 7.28) 0.66
Huang (2017) 2.02 (0.18, 22.71) 0.22
Huang (2007) 1.65 (0.69, 3.90) 1.77
Huang (2005a) 1.24 (0.47, 3.24) 1.65
Huang (2005b) 0.76 (0.36, 1.60) 3.74
Inoue (2003) 0.15 (0.01, 2.68) 1.05
Kiczmer (2018) 7.02 (2.27, 21.72) 0.44
Kietthubthew (2006) 1.55 (0.10, 25.09) 0.17
Li (2005) 0.40 (0.04, 3.92) 0.60
Liu (2006) 3.32 (0.16, 70.37) 0.13
Liu (2009) 1.13 (0.40, 3.14) 1.55
Loh (2011) 2.16 (0.77, 6.06) 0.88
Lu (2006) 0.87 (0.24, 3.14) 1.14
McKean (2009) 1.13 (0.65, 1.97) 5.19
O'Mara (2011) 1.14 (0.62, 2.10) 4.32
O'Mara (2011) 1.62 (0.62, 4.23) 1.51
Palli (2010) 0.67 (0.21, 2.11) 1.71
Rajaraman (2010) 1.02 (0.42, 2.45) 2.23
Rajaraman (2010) 1.24 (0.39, 3.91) 1.11
Rajaraman (2010) 1.21 (0.26, 5.53) 0.64
Ritchey (2005) 3.08 (0.28, 34.27) 0.18
Shah (2012) 4.28 (0.20, 90.54) 0.12
Shen (2005) 1.09 (0.59, 2.03) 4.32
Shen (2007) 0.81 (0.36, 1.86) 2.79
Shi (2011) 1.38 (0.31, 6.18) 0.63
Stern (2007) 0.30 (0.04, 2.34) 1.17
Tranah (2006) 2.19 (0.90, 5.31) 1.11
Tranah (2006) 1.69 (0.54, 5.28) 0.99
Wang (2006) 1.66 (0.93, 2.96) 4.08
Yang (2009) 1.48 (0.17, 12.91) 0.26
Zhang (2008) 0.27 (0.03, 2.23) 1.07
Zhang (2010) 0.70 (0.29, 1.70) 2.79
Zienolddiny (2006) 1.58 (0.68, 3.65) 1.96
Huang (2010) (Excluded) 0.00
Liu (2002a) (Excluded) 0.00
Liu (2002b) (Excluded) 0.00
Liu (2002b) (Excluded) 0.00
Overall (I-squared = 3.2%, p = 0.410) 1.32 (1.17, 1.49) 100.00
.00887
1
113

Figure S3

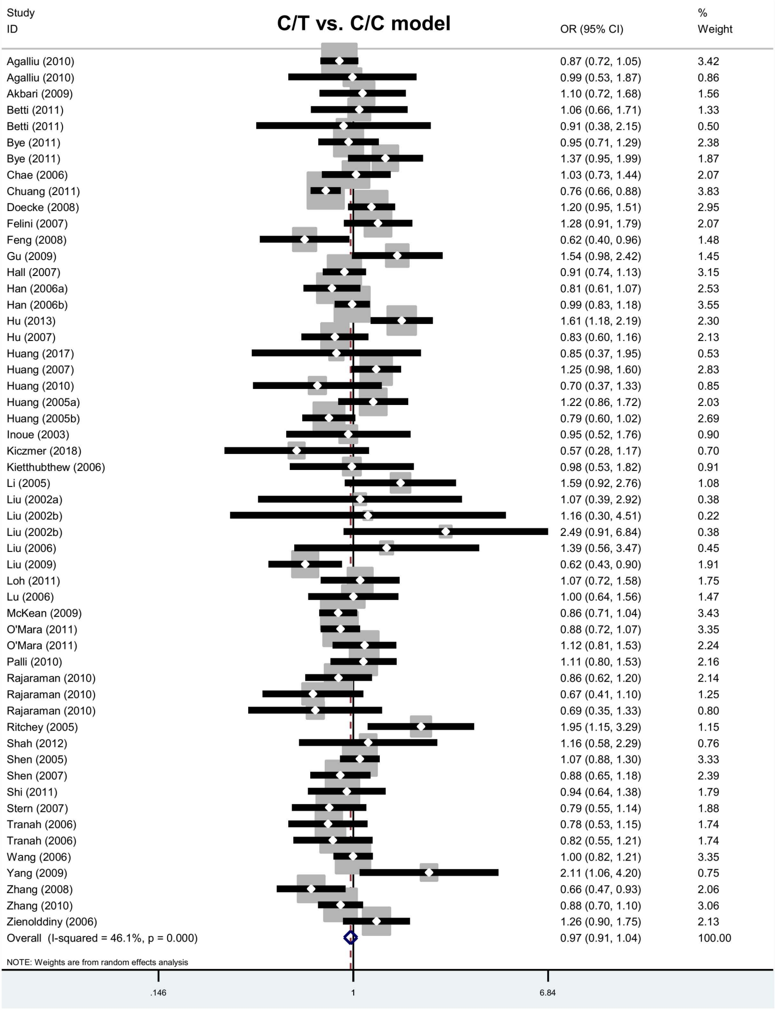

Study
ID
C/T vs. C/C model
OR (95% CI)
%
Weight
Agalliu (2010) 0.87 (0.72, 1.05) 3.42
Agalliu (2010) 0.99 (0.53, 1.87) 0.86
Akbari (2009) 1.10 (0.72, 1.68) 1.56
Betti (2011) 1.06 (0.66, 1.71) 1.33
Betti (2011) 0.91 (0.38, 2.15) 0.50
Bye (2011) 0.95 (0.71, 1.29) 2.38
Bye (2011) 1.37 (0.95, 1.99) 1.87
Chae (2006) 1.03 (0.73, 1.44) 2.07
Chuang (2011) 0.76 (0.66, 0.88) 3.83
Doecke (2008) 1.20 (0.95, 1.51) 2.95
Felini (2007) 1.28 (0.91, 1.79) 2.07
Feng (2008) 0.62 (0.40, 0.96) 1.48
Gu (2009) 1.54 (0.98, 2.42) 1.45
Hall (2007) 0.91 (0.74, 1.13) 3.15
Han (2006a) 0.81 (0.61, 1.07) 2.53
Han (2006b) 0.99 (0.83, 1.18) 3.55
Hu (2013) 1.61 (1.18, 2.19) 2.30
Hu (2007) 0.83 (0.60, 1.16) 2.13
Huang (2017) 0.85 (0.37, 1.95) 0.53
Huang (2007) 1.25 (0.98, 1.60) 2.83
Huang (2010) 0.70 (0.37, 1.33) 0.85
Huang (2005a) 1.22 (0.86, 1.72) 2.03
Huang (2005b) 0.79 (0.60, 1.02) 2.69
Inoue (2003) 0.95 (0.52, 1.76) 0.90
Kiczmer (2018) 0.57 (0.28, 1.17) 0.70
Kietthubthew (2006) 0.98 (0.53, 1.82) 0.91
Li (2005) 1.59 (0.92, 2.76) 1.08
Liu (2002a) 1.07 (0.39, 2.92) 0.38
Liu (2002b) 1.16 (0.30, 4.51) 0.22
Liu (2002b) 2.49 (0.91, 6.84) 0.38
Liu (2006) 1.39 (0.56, 3.47) 0.45
Liu (2009) 0.62 (0.43, 0.90) 1.91
Loh (2011) 1.07 (0.72, 1.58) 1.75
Lu (2006) 1.00 (0.64, 1.56) 1.47
McKean (2009) 0.86 (0.71, 1.04) 3.43
O'Mara (2011) 0.88 (0.72, 1.07) 3.35
O'Mara (2011) 1.12 (0.81, 1.53) 2.24
Palli (2010) 1.11 (0.80, 1.53) 2.16
Rajaraman (2010) 0.86 (0.62, 1.20) 2.14
Rajaraman (2010) 0.67 (0.41, 1.10) 1.25
Rajaraman (2010) 0.69 (0.35, 1.33) 0.80
Ritchey (2005) 1.95 (1.15, 3.29) 1.15
Shah (2012) 1.16 (0.58, 2.29) 0.76
Shen (2005) 1.07 (0.88, 1.30) 3.33
Shen (2007) 0.88 (0.65, 1.18) 2.39
Shi (2011) 0.94 (0.64, 1.38) 1.79
Stern (2007) 0.79 (0.55, 1.14) 1.88
Tranah (2006) 0.78 (0.53, 1.15) 1.74
Tranah (2006) 0.82 (0.55, 1.21) 1.74
Wang (2006) 1.00 (0.82, 1.21) 3.35
Yang (2009) 2.11 (1.06, 4.20) 0.75
Zhang (2008) 0.66 (0.47, 0.93) 2.06
Zhang (2010) 0.88 (0.70, 1.10) 3.06
Zienolddiny (2006) 1.26 (0.90, 1.75) 2.13
Overall (I-squared = 46.1%, p = 0.000) 0.97 (0.91, 1.04) 100.00
NOTE: Weights are from random effects analysis
.146
1
6.84

Figure S4

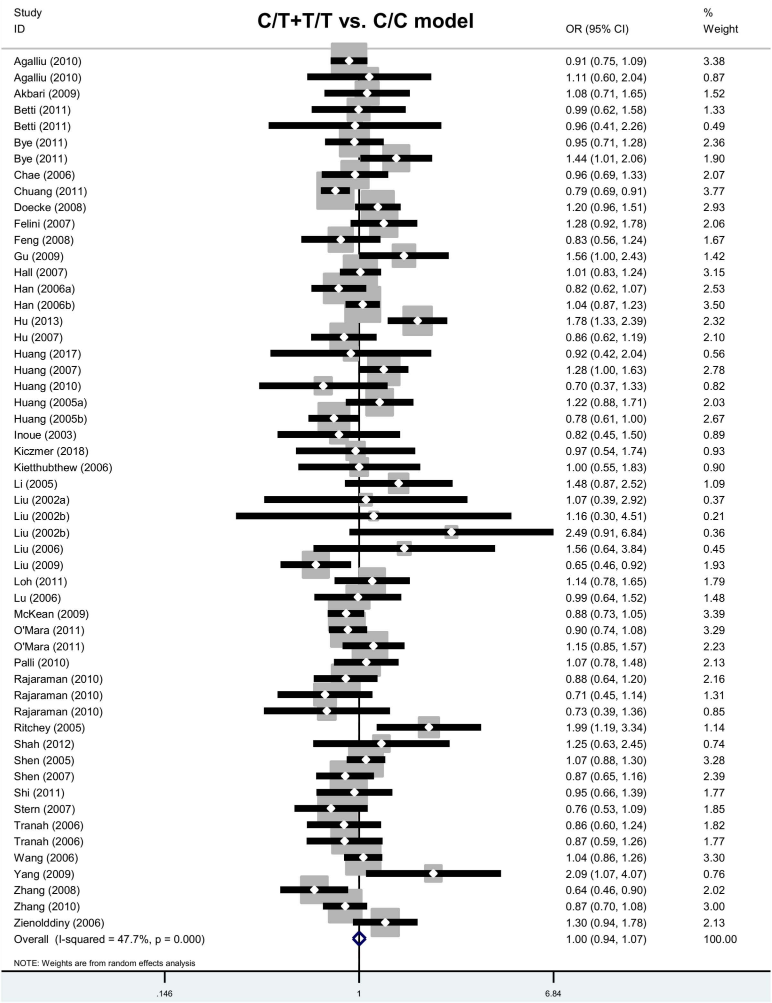
C/T+T/T vs. C/C model
Study ID
OR (95% CI)
% Weight
Agalliu (2010) 0.91 (0.75, 1.09) 3.38
Agalliu (2010) 1.11 (0.60, 2.04) 0.87
Akbari (2009) 1.08 (0.71, 1.65) 1.52
Betti (2011) 0.99 (0.62, 1.58) 1.33
Betti (2011) 0.96 (0.41, 2.26) 0.49
Bye (2011) 0.95 (0.71, 1.28) 2.36
Bye (2011) 1.44 (1.01, 2.06) 1.90
Chae (2006) 0.96 (0.69, 1.33) 2.07
Chuang (2011) 0.79 (0.69, 0.91) 3.77
Doecke (2008) 1.20 (0.96, 1.51) 2.93
Felini (2007) 1.28 (0.92, 1.78) 2.06
Feng (2008) 0.83 (0.56, 1.24) 1.67
Gu (2009) 1.56 (1.00, 2.43) 1.42
Hall (2007) 1.01 (0.83, 1.24) 3.15
Han (2006a) 0.82 (0.62, 1.07) 2.53
Han (2006b) 1.04 (0.87, 1.23) 3.50
Hu (2013) 1.78 (1.33, 2.39) 2.32
Hu (2007) 0.86 (0.62, 1.19) 2.10
Huang (2017) 0.92 (0.42, 2.04) 0.56
Huang (2007) 1.28 (1.00, 1.63) 2.78
Huang (2010) 0.70 (0.37, 1.33) 0.82
Huang (2005a) 1.22 (0.88, 1.71) 2.03
Huang (2005b) 0.78 (0.61, 1.00) 2.67
Inoue (2003) 0.82 (0.45, 1.50) 0.89
Kiczmer (2018) 0.97 (0.54, 1.74) 0.93
Kietthubthew (2006) 1.00 (0.55, 1.83) 0.90
Li (2005) 1.48 (0.87, 2.52) 1.09
Liu (2002a) 1.07 (0.39, 2.92) 0.37
Liu (2002b) 1.16 (0.30, 4.51) 0.21
Liu (2002b) 2.49 (0.91, 6.84) 0.36
Liu (2006) 1.56 (0.64, 3.84) 0.45
Liu (2009) 0.65 (0.46, 0.92) 1.93
Loh (2011) 1.14 (0.78, 1.65) 1.79
Lu (2006) 0.99 (0.64, 1.52) 1.48
McKean (2009) 0.88 (0.73, 1.05) 3.39
O'Mara (2011) 0.90 (0.74, 1.08) 3.29
O'Mara (2011) 1.15 (0.85, 1.57) 2.23
Palli (2010) 1.07 (0.78, 1.48) 2.13
Rajaraman (2010) 0.88 (0.64, 1.20) 2.16
Rajaraman (2010) 0.71 (0.45, 1.14) 1.31
Rajaraman (2010) 0.73 (0.39, 1.36) 0.85
Ritchey (2005) 1.99 (1.19, 3.34) 1.14
Shah (2012) 1.25 (0.63, 2.45) 0.74
Shen (2005) 1.07 (0.88, 1.30) 3.28
Shen (2007) 0.87 (0.65, 1.16) 2.39
Shi (2011) 0.95 (0.66, 1.39) 1.77
Stern (2007) 0.76 (0.53, 1.09) 1.85
Tranah (2006) 0.86 (0.60, 1.24) 1.82
Tranah (2006) 0.87 (0.59, 1.26) 1.77
Wang (2006) 1.04 (0.86, 1.26) 3.30
Yang (2009) 2.09 (1.07, 4.07) 0.76
Zhang (2008) 0.64 (0.46, 0.90) 2.02
Zhang (2010) 0.87 (0.70, 1.08) 3.00
Zienolddiny (2006) 1.30 (0.94, 1.78) 2.13
Overall (I-squared = 47.7%, p = 0.000) 1.00 (0.94, 1.07) 100.00
NOTE: Weights are from random effects analysis
.146
1
6.84

Figure S5

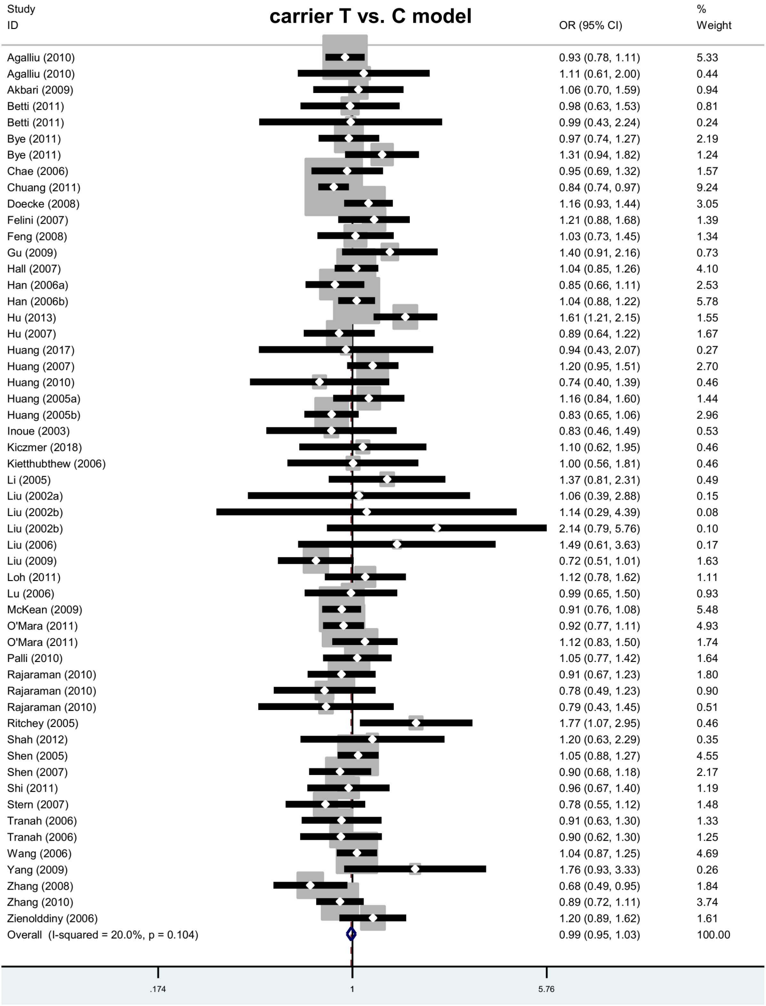
carrier T vs. C model
Study ID
OR (95% CI)
% Weight
Agalliu (2010) 0.93 (0.78, 1.11) 5.33
Agalliu (2010) 1.11 (0.61, 2.00) 0.44
Akbari (2009) 1.06 (0.70, 1.59) 0.94
Betti (2011) 0.98 (0.63, 1.53) 0.81
Betti (2011) 0.99 (0.43, 2.24) 0.24
Bye (2011) 0.97 (0.74, 1.27) 2.19
Bye (2011) 1.31 (0.94, 1.82) 1.24
Chae (2006) 0.95 (0.69, 1.32) 1.57
Chuang (2011) 0.84 (0.74, 0.97) 9.24
Doecke (2008) 1.16 (0.93, 1.44) 3.05
Felini (2007) 1.21 (0.88, 1.68) 1.39
Feng (2008) 1.03 (0.73, 1.45) 1.34
Gu (2009) 1.40 (0.91, 2.16) 0.73
Hall (2007) 1.04 (0.85, 1.26) 4.10
Han (2006a) 0.85 (0.66, 1.11) 2.53
Han (2006b) 1.04 (0.88, 1.22) 5.78
Hu (2013) 1.61 (1.21, 2.15) 1.55
Hu (2007) 0.89 (0.64, 1.22) 1.67
Huang (2017) 0.94 (0.43, 2.07) 0.27
Huang (2007) 1.20 (0.95, 1.51) 2.70
Huang (2010) 0.74 (0.40, 1.39) 0.46
Huang (2005a) 1.16 (0.84, 1.60) 1.44
Huang (2005b) 0.83 (0.65, 1.06) 2.96
Inoue (2003) 0.83 (0.46, 1.49) 0.53
Kiczmer (2018) 1.10 (0.62, 1.95) 0.46
Kietthubthew (2006) 1.00 (0.56, 1.81) 0.46
Li (2005) 1.37 (0.81, 2.31) 0.49
Liu (2002a) 1.06 (0.39, 2.88) 0.15
Liu (2002b) 1.14 (0.29, 4.39) 0.08
Liu (2002b) 2.14 (0.79, 5.76) 0.10
Liu (2006) 1.49 (0.61, 3.63) 0.17
Liu (2009) 0.72 (0.51, 1.01) 1.63
Loh (2011) 1.12 (0.78, 1.62) 1.11
Lu (2006) 0.99 (0.65, 1.50) 0.93
McKean (2009) 0.91 (0.76, 1.08) 5.48
O'Mara (2011) 0.92 (0.77, 1.11) 4.93
O'Mara (2011) 1.12 (0.83, 1.50) 1.74
Palli (2010) 1.05 (0.77, 1.42) 1.64
Rajaraman (2010) 0.91 (0.67, 1.23) 1.80
Rajaraman (2010) 0.78 (0.49, 1.23) 0.90
Rajaraman (2010) 0.79 (0.43, 1.45) 0.51
Ritchey (2005) 1.77 (1.07, 2.95) 0.46
Shah (2012) 1.20 (0.63, 2.29) 0.35
Shen (2005) 1.05 (0.88, 1.27) 4.55
Shen (2007) 0.90 (0.68, 1.18) 2.17
Shi (2011) 0.96 (0.67, 1.40) 1.19
Stern (2007) 0.78 (0.55, 1.12) 1.48
Tranah (2006) 0.91 (0.63, 1.30) 1.33
Tranah (2006) 0.90 (0.62, 1.30) 1.25
Wang (2006) 1.04 (0.87, 1.25) 4.69
Yang (2009) 1.76 (0.93, 3.33) 0.26
Zhang (2008) 0.68 (0.49, 0.95) 1.84
Zhang (2010) 0.89 (0.72, 1.11) 3.74
Zienolddiny (2006) 1.20 (0.89, 1.62) 1.61
Overall (I-squared = 20.0%, p = 0.104) 0.99 (0.95, 1.03) 100.00
.174
1
5.76

Figure S6

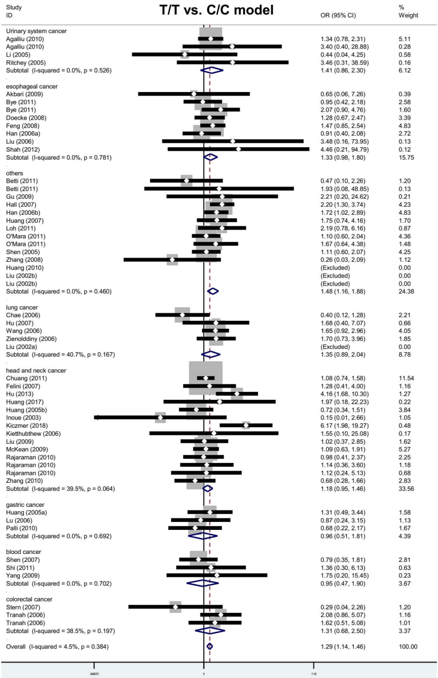
T/T vs. C/C model
Study ID
OR (95% CI)
% Weight
Urinary system cancer
Agalliu (2010) 1.34 (0.78, 2.31) 5.11
Agalliu (2010) 3.40 (0.40, 28.88) 0.28
Li (2005) 0.44 (0.04, 4.25) 0.58
Ritchey (2005) 3.46 (0.31, 38.59) 0.16
Subtotal (I-squared = 0.0%, p = 0.526) 1.41 (0.86, 2.30) 6.12
esophageal cancer
Akbari (2009) 0.65 (0.06, 7.26) 0.39
Bye (2011) 0.95 (0.42, 2.18) 2.58
Bye (2011) 2.07 (0.90, 4.76) 1.60
Doecke (2008) 1.28 (0.67, 2.47) 3.39
Feng (2008) 1.47 (0.85, 2.54) 4.83
Han (2006a) 0.91 (0.40, 2.08) 2.72
Liu (2006) 3.48 (0.16, 73.95) 0.13
Shah (2012) 4.46 (0.21, 94.79) 0.12
Subtotal (I-squared = 0.0%, p = 0.781) 1.33 (0.98, 1.80) 15.75
others
Betti (2011) 0.47 (0.10, 2.26) 1.20
Betti (2011) 1.93 (0.08, 48.85) 0.13
Gu (2009) 2.21 (0.20, 24.62) 0.21
Hall (2007) 2.20 (1.30, 3.74) 4.23
Han (2006b) 1.72 (1.02, 2.89) 4.83
Huang (2007) 1.75 (0.74, 4.16) 1.70
Loh (2011) 2.19 (0.78, 6.16) 0.87
O'Mara (2011) 1.10 (0.60, 2.04) 4.36
O'Mara (2011) 1.67 (0.64, 4.38) 1.48
Shen (2005) 1.11 (0.60, 2.07) 4.25
Zhang (2008) 0.26 (0.03, 2.09) 1.12
Huang (2010) (Excluded) 0.00
Liu (2002b) (Excluded) 0.00
Liu (2002b) (Excluded) 0.00
Subtotal (I-squared = 0.0%, p = 0.460) 1.48 (1.16, 1.88) 24.38
lung cancer
Chae (2006) 0.40 (0.12, 1.28) 2.21
Hu (2007) 1.68 (0.40, 7.07) 0.66
Wang (2006) 1.65 (0.92, 2.96) 4.05
Zienolddiny (2006) 1.70 (0.73, 3.96) 1.85
Liu (2002a) (Excluded) 0.00
Subtotal (I-squared = 40.7%, p = 0.167) 1.35 (0.89, 2.04) 8.78
head and neck cancer
Chuang (2011) 1.08 (0.74, 1.58) 11.54
Felini (2007) 1.28 (0.41, 4.00) 1.16
Hu (2013) 4.16 (1.68, 10.30) 1.27
Huang (2017) 1.97 (0.18, 22.23) 0.22
Huang (2005b) 0.72 (0.34, 1.51) 3.84
Inoue (2003) 0.15 (0.01, 2.66) 1.05
Kiczmer (2018) 6.17 (1.98, 19.27) 0.48
Kietthubthew (2006) 1.55 (0.10, 25.08) 0.17
Liu (2009) 1.02 (0.37, 2.85) 1.62
McKean (2009) 1.09 (0.63, 1.91) 5.27
Rajaraman (2010) 0.98 (0.41, 2.37) 2.25
Rajaraman (2010) 1.14 (0.36, 3.60) 1.18
Rajaraman (2010) 1.12 (0.24, 5.13) 0.68
Zhang (2010) 0.68 (0.28, 1.66) 2.83
Subtotal (I-squared = 39.5%, p = 0.064) 1.18 (0.95, 1.46) 33.56
gastric cancer
Huang (2005a) 1.31 (0.49, 3.44) 1.58
Lu (2006) 0.87 (0.24, 3.15) 1.13
Palli (2010) 0.68 (0.22, 2.17) 1.67
Subtotal (I-squared = 0.0%, p = 0.692) 0.96 (0.51, 1.81) 4.39
blood cancer
Shen (2007) 0.79 (0.35, 1.81) 2.81
Shi (2011) 1.36 (0.30, 6.13) 0.63
Yang (2009) 1.75 (0.20, 15.45) 0.23
Subtotal (I-squared = 0.0%, p = 0.702) 0.95 (0.47, 1.90) 3.67
colorectal cancer
Stern (2007) 0.29 (0.04, 2.26) 1.20
Tranah (2006) 2.08 (0.86, 5.07) 1.16
Tranah (2006) 1.62 (0.51, 5.08) 1.01
Subtotal (I-squared = 38.5%, p = 0.197) 1.31 (0.68, 2.50) 3.37
Overall (I-squared = 4.5%, p = 0.384) 1.29 (1.14, 1.46) 100.00
.00872
1
115

Figure S7

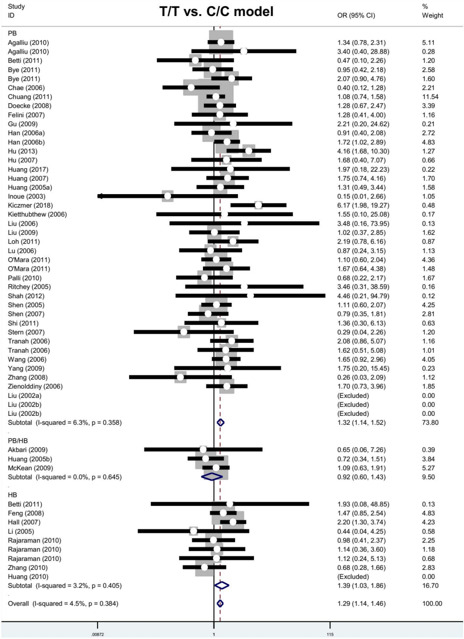

Study ID
T/T vs. C/C model
OR (95% CI)
% Weight
PB
Agalliu (2010) 1.34 (0.78, 2.31) 5.11
Agalliu (2010) 3.40 (0.40, 28.88) 0.28
Betti (2011) 0.47 (0.10, 2.26) 1.20
Bye (2011) 0.95 (0.42, 2.18) 2.58
Bye (2011) 2.07 (0.90, 4.76) 1.60
Chae (2006) 0.40 (0.12, 1.28) 2.21
Chuang (2011) 1.08 (0.74, 1.58) 11.54
Doecke (2008) 1.28 (0.67, 2.47) 3.39
Felini (2007) 1.28 (0.41, 4.00) 1.16
Gu (2009) 2.21 (0.20, 24.62) 0.21
Han (2006a) 0.91 (0.40, 2.08) 2.72
Han (2006b) 1.72 (1.02, 2.89) 4.83
Hu (2013) 4.16 (1.68, 10.30) 1.27
Hu (2007) 1.68 (0.40, 7.07) 0.66
Huang (2017) 1.97 (0.18, 22.23) 0.22
Huang (2007) 1.75 (0.74, 4.16) 1.70
Huang (2005a) 1.31 (0.49, 3.44) 1.58
Inoue (2003) 0.15 (0.01, 2.66) 1.05
Kiczmer (2018) 6.17 (1.98, 19.27) 0.48
Kietthubthew (2006) 1.55 (0.10, 25.08) 0.17
Liu (2006) 3.48 (0.16, 73.95) 0.13
Liu (2009) 1.02 (0.37, 2.85) 1.62
Loh (2011) 2.19 (0.78, 6.16) 0.87
Lu (2006) 0.87 (0.24, 3.15) 1.13
O'Mara (2011) 1.10 (0.60, 2.04) 4.36
O'Mara (2011) 1.67 (0.64, 4.38) 1.48
Palli (2010) 0.68 (0.22, 2.17) 1.67
Ritchey (2005) 3.46 (0.31, 38.59) 0.16
Shah (2012) 4.46 (0.21, 94.79) 0.12
Shen (2005) 1.11 (0.60, 2.07) 4.25
Shen (2007) 0.79 (0.35, 1.81) 2.81
Shi (2011) 1.36 (0.30, 6.13) 0.63
Stern (2007) 0.29 (0.04, 2.26) 1.20
Tranah (2006) 2.08 (0.86, 5.07) 1.16
Tranah (2006) 1.62 (0.51, 5.08) 1.01
Wang (2006) 1.65 (0.92, 2.96) 4.05
Yang (2009) 1.75 (0.20, 15.45) 0.23
Zhang (2008) 0.26 (0.03, 2.09) 1.12
Zienolddiny (2006) 1.70 (0.73, 3.96) 1.85
Liu (2002a) (Excluded) 0.00
Liu (2002b) (Excluded) 0.00
Liu (2002b) (Excluded) 0.00
Subtotal (I-squared = 6.3%, p = 0.358) 1.32 (1.14, 1.52) 73.80
PB/HB
Akbari (2009) 0.65 (0.06, 7.26) 0.39
Huang (2005b) 0.72 (0.34, 1.51) 3.84
McKean (2009) 1.09 (0.63, 1.91) 5.27
Subtotal (I-squared = 0.0%, p = 0.645) 0.92 (0.60, 1.43) 9.50
HB
Betti (2011) 1.93 (0.08, 48.85) 0.13
Feng (2008) 1.47 (0.85, 2.54) 4.83
Hall (2007) 2.20 (1.30, 3.74) 4.23
Li (2005) 0.44 (0.04, 4.25) 0.58
Rajaraman (2010) 0.98 (0.41, 2.37) 2.25
Rajaraman (2010) 1.14 (0.36, 3.60) 1.18
Rajaraman (2010) 1.12 (0.24, 5.13) 0.68
Zhang (2010) 0.68 (0.28, 1.66) 2.83
Huang (2010) (Excluded) 0.00
Subtotal (I-squared = 3.2%, p = 0.405) 1.39 (1.03, 1.86) 16.70
Overall (I-squared = 4.5%, p = 0.384) 1.29 (1.14, 1.46) 100.00
.00872
1
115

Figure S8

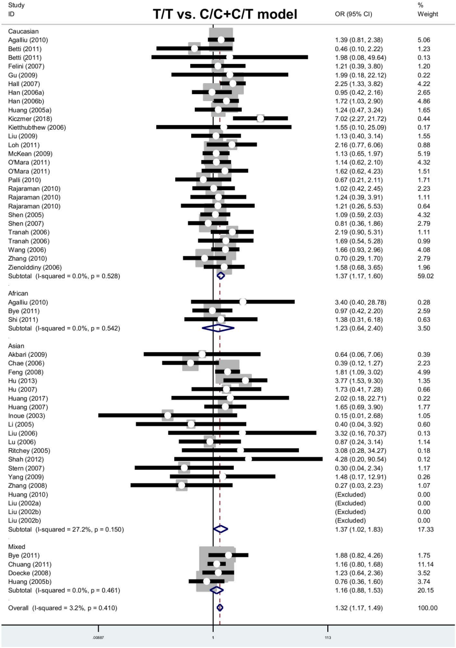

T/T vs. C/C+C/T model
Study ID
OR (95% CI)
% Weight
Caucasian
Agalliu (2010) 1.39 (0.81, 2.38) 5.06
Betti (2011) 0.46 (0.10, 2.22) 1.23
Betti (2011) 1.98 (0.08, 49.64) 0.13
Felini (2007) 1.21 (0.39, 3.80) 1.20
Gu (2009) 1.99 (0.18, 22.12) 0.22
Hall (2007) 2.25 (1.33, 3.82) 4.22
Han (2006a) 0.95 (0.42, 2.16) 2.65
Han (2006b) 1.72 (1.03, 2.90) 4.86
Huang (2005a) 1.24 (0.47, 3.24) 1.65
Kiczmer (2018) 7.02 (2.27, 21.72) 0.44
Kietthubthew (2006) 1.55 (0.10, 25.09) 0.17
Liu (2009) 1.13 (0.40, 3.14) 1.55
Loh (2011) 2.16 (0.77, 6.06) 0.88
McKean (2009) 1.13 (0.65, 1.97) 5.19
O'Mara (2011) 1.14 (0.62, 2.10) 4.32
O'Mara (2011) 1.62 (0.62, 4.23) 1.51
Palli (2010) 0.67 (0.21, 2.11) 1.71
Rajaraman (2010) 1.02 (0.42, 2.45) 2.23
Rajaraman (2010) 1.24 (0.39, 3.91) 1.11
Rajaraman (2010) 1.21 (0.26, 5.53) 0.64
Shen (2005) 1.09 (0.59, 2.03) 4.32
Shen (2007) 0.81 (0.36, 1.86) 2.79
Tranah (2006) 2.19 (0.90, 5.31) 1.11
Tranah (2006) 1.69 (0.54, 5.28) 0.99
Wang (2006) 1.66 (0.93, 2.96) 4.08
Zhang (2010) 0.70 (0.29, 1.70) 2.79
Zienolddiny (2006) 1.58 (0.68, 3.65) 1.96
Subtotal (I-squared = 0.0%, p = 0.528) 1.37 (1.17, 1.60) 59.02
African
Agalliu (2010) 3.40 (0.40, 28.78) 0.28
Bye (2011) 0.97 (0.42, 2.20) 2.59
Shi (2011) 1.38 (0.31, 6.18) 0.63
Subtotal (I-squared = 0.0%, p = 0.542) 1.23 (0.64, 2.40) 3.50
Asian
Akbari (2009) 0.64 (0.06, 7.06) 0.39
Chae (2006) 0.39 (0.12, 1.27) 2.23
Feng (2008) 1.81 (1.09, 3.02) 4.99
Hu (2013) 3.77 (1.53, 9.30) 1.35
Hu (2007) 1.73 (0.41, 7.28) 0.66
Huang (2017) 2.02 (0.18, 22.71) 0.22
Huang (2007) 1.65 (0.69, 3.90) 1.77
Inoue (2003) 0.15 (0.01, 2.68) 1.05
Li (2005) 0.40 (0.04, 3.92) 0.60
Liu (2006) 3.32 (0.16, 70.37) 0.13
Lu (2006) 0.87 (0.24, 3.14) 1.14
Ritchey (2005) 3.08 (0.28, 34.27) 0.18
Shah (2012) 4.28 (0.20, 90.54) 0.12
Stern (2007) 0.30 (0.04, 2.34) 1.17
Yang (2009) 1.48 (0.17, 12.91) 0.26
Zhang (2008) 0.27 (0.03, 2.23) 1.07
Huang (2010) (Excluded) 0.00
Liu (2002a) (Excluded) 0.00
Liu (2002b) (Excluded) 0.00
Liu (2002b) (Excluded) 0.00
Subtotal (I-squared = 27.2%, p = 0.150) 1.37 (1.02, 1.83) 17.33
Mixed
Bye (2011) 1.88 (0.82, 4.26) 1.75
Chuang (2011) 1.16 (0.80, 1.68) 11.14
Doecke (2008) 1.23 (0.64, 2.36) 3.52
Huang (2005b) 0.76 (0.36, 1.60) 3.74
Subtotal (I-squared = 0.0%, p = 0.461) 1.16 (0.88, 1.53) 20.15
Overall (I-squared = 3.2%, p = 0.410) 1.32 (1.17, 1.49) 100.00
.00887
1
113

Figure S9

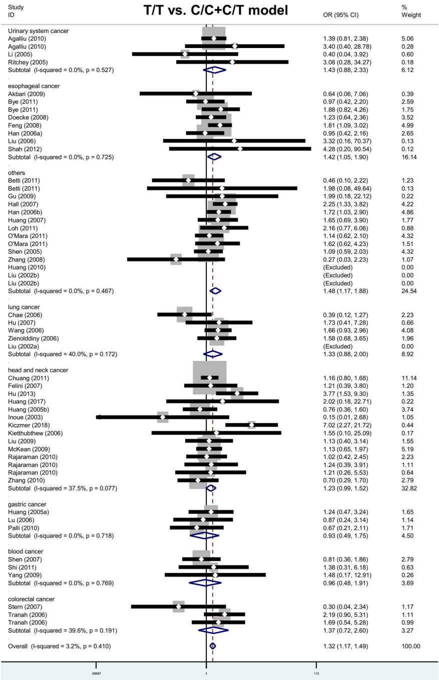

Study
ID
T/T vs. C/C+C/T model
OR (95% CI)
%
Weight
Urinary system cancer
Agalliu (2010) 1.39 (0.81, 2.38) 5.06
Agalliu (2010) 3.40 (0.40, 28.78) 0.28
Li (2005) 0.40 (0.04, 3.92) 0.60
Ritchey (2005) 3.08 (0.28, 34.27) 0.18
Subtotal (I-squared = 0.0%, p = 0.527) 1.43 (0.88, 2.33) 6.12
esophageal cancer
Akbari (2009) 0.64 (0.06, 7.06) 0.39
Bye (2011) 0.97 (0.42, 2.20) 2.59
Bye (2011) 1.88 (0.82, 4.26) 1.75
Doecke (2008) 1.23 (0.64, 2.36) 3.52
Feng (2008) 1.81 (1.09, 3.02) 4.99
Han (2006a) 0.95 (0.42, 2.16) 2.65
Liu (2006) 3.32 (0.16, 70.37) 0.13
Shah (2012) 4.28 (0.20, 90.54) 0.12
Subtotal (I-squared = 0.0%, p = 0.725) 1.42 (1.05, 1.90) 16.14
others
Betti (2011) 0.46 (0.10, 2.22) 1.23
Betti (2011) 1.98 (0.08, 49.64) 0.13
Gu (2009) 1.99 (0.18, 22.12) 0.22
Hall (2007) 2.25 (1.33, 3.82) 4.22
Han (2006b) 1.72 (1.03, 2.90) 4.86
Huang (2007) 1.65 (0.69, 3.90) 1.77
Loh (2011) 2.16 (0.77, 6.06) 0.88
O'Mara (2011) 1.14 (0.62, 2.10) 4.32
O'Mara (2011) 1.62 (0.62, 4.23) 1.51
Shen (2005) 1.09 (0.59, 2.03) 4.32
Zhang (2008) 0.27 (0.03, 2.23) 1.07
Huang (2010) (Excluded) 0.00
Liu (2002b) (Excluded) 0.00
Liu (2002b) (Excluded) 0.00
Subtotal (I-squared = 0.0%, p = 0.467) 1.48 (1.17, 1.88) 24.54
lung cancer
Chae (2006) 0.39 (0.12, 1.27) 2.23
Hu (2007) 1.73 (0.41, 7.28) 0.66
Wang (2006) 1.66 (0.93, 2.96) 4.08
Zienolddiny (2006) 1.58 (0.68, 3.65) 1.96
Liu (2002a) (Excluded) 0.00
Subtotal (I-squared = 40.0%, p = 0.172) 1.33 (0.88, 2.00) 8.92
head and neck cancer
Chuang (2011) 1.16 (0.80, 1.68) 11.14
Felini (2007) 1.21 (0.39, 3.80) 1.20
Hu (2013) 3.77 (1.53, 9.30) 1.35
Huang (2017) 2.02 (0.18, 22.71) 0.22
Huang (2005b) 0.76 (0.36, 1.60) 3.74
Inoue (2003) 0.15 (0.01, 2.68) 1.05
Kiczmer (2018) 7.02 (2.27, 21.72) 0.44
Kietthubthew (2006) 1.55 (0.10, 25.09) 0.17
Liu (2009) 1.13 (0.40, 3.14) 1.55
McKean (2009) 1.13 (0.65, 1.97) 5.19
Rajaraman (2010) 1.02 (0.42, 2.45) 2.23
Rajaraman (2010) 1.24 (0.39, 3.91) 1.11
Rajaraman (2010) 1.21 (0.26, 5.53) 0.64
Zhang (2010) 0.70 (0.29, 1.70) 2.79
Subtotal (I-squared = 37.5%, p = 0.077) 1.23 (0.99, 1.52) 32.82
gastric cancer
Huang (2005a) 1.24 (0.47, 3.24) 1.65
Lu (2006) 0.87 (0.24, 3.14) 1.14
Palli (2010) 0.67 (0.21, 2.11) 1.71
Subtotal (I-squared = 0.0%, p = 0.718) 0.93 (0.49, 1.75) 4.50
blood cancer
Shen (2007) 0.81 (0.36, 1.86) 2.79
Shi (2011) 1.38 (0.31, 6.18) 0.63
Yang (2009) 1.48 (0.17, 12.91) 0.26
Subtotal (I-squared = 0.0%, p = 0.769) 0.96 (0.48, 1.91) 3.69
colorectal cancer
Stern (2007) 0.30 (0.04, 2.34) 1.17
Tranah (2006) 2.19 (0.90, 5.31) 1.11
Tranah (2006) 1.69 (0.54, 5.28) 0.99
Subtotal (I-squared = 39.6%, p = 0.191) 1.37 (0.72, 2.60) 3.27
Overall (I-squared = 3.2%, p = 0.410) 1.32 (1.17, 1.49) 100.00
.00887
1
113

Figure S10

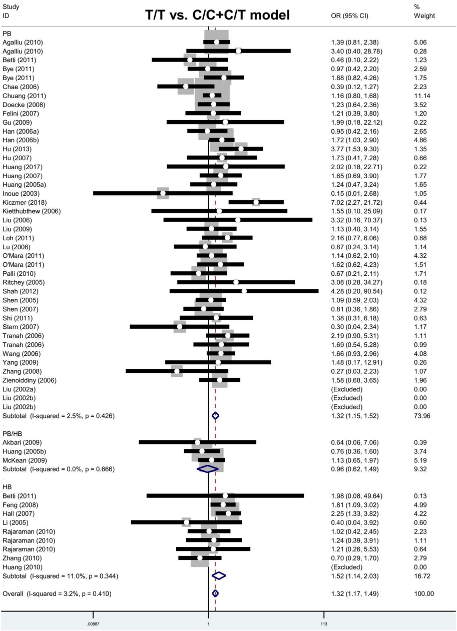

Study ID
T/T vs. C/C+C/T model
OR (95% CI)
% Weight
PB
Agalliu (2010) 1.39 (0.81, 2.38) 5.06
Agalliu (2010) 3.40 (0.40, 28.78) 0.28
Betti (2011) 0.46 (0.10, 2.22) 1.23
Bye (2011) 0.97 (0.42, 2.20) 2.59
Bye (2011) 1.88 (0.82, 4.26) 1.75
Chae (2006) 0.39 (0.12, 1.27) 2.23
Chuang (2011) 1.16 (0.80, 1.68) 11.14
Doecke (2008) 1.23 (0.64, 2.36) 3.52
Felini (2007) 1.21 (0.39, 3.80) 1.20
Gu (2009) 1.99 (0.18, 22.12) 0.22
Han (2006a) 0.95 (0.42, 2.16) 2.65
Han (2006b) 1.72 (1.03, 2.90) 4.86
Hu (2013) 3.77 (1.53, 9.30) 1.35
Hu (2007) 1.73 (0.41, 7.28) 0.66
Huang (2017) 2.02 (0.18, 22.71) 0.22
Huang (2007) 1.65 (0.69, 3.90) 1.77
Huang (2005a) 1.24 (0.47, 3.24) 1.65
Inoue (2003) 0.15 (0.01, 2.68) 1.05
Kiczmer (2018) 7.02 (2.27, 21.72) 0.44
Kietthubthew (2006) 1.55 (0.10, 25.09) 0.17
Liu (2006) 3.32 (0.16, 70.37) 0.13
Liu (2009) 1.13 (0.40, 3.14) 1.55
Loh (2011) 2.16 (0.77, 6.06) 0.88
Lu (2006) 0.87 (0.24, 3.14) 1.14
O'Mara (2011) 1.14 (0.62, 2.10) 4.32
O'Mara (2011) 1.62 (0.62, 4.23) 1.51
Palli (2010) 0.67 (0.21, 2.11) 1.71
Ritchey (2005) 3.08 (0.28, 34.27) 0.18
Shah (2012) 4.28 (0.20, 90.54) 0.12
Shen (2005) 1.09 (0.59, 2.03) 4.32
Shen (2007) 0.81 (0.36, 1.86) 2.79
Shi (2011) 1.38 (0.31, 6.18) 0.63
Stern (2007) 0.30 (0.04, 2.34) 1.17
Tranah (2006) 2.19 (0.90, 5.31) 1.11
Tranah (2006) 1.69 (0.54, 5.28) 0.99
Wang (2006) 1.66 (0.93, 2.96) 4.08
Yang (2009) 1.48 (0.17, 12.91) 0.26
Zhang (2008) 0.27 (0.03, 2.23) 1.07
Zienolddiny (2006) 1.58 (0.68, 3.65) 1.96
Liu (2002a) (Excluded) 0.00
Liu (2002b) (Excluded) 0.00
Liu (2002b) (Excluded) 0.00
Subtotal (I-squared = 2.5%, p = 0.426) 1.32 (1.15, 1.52) 73.96
.
PB/HB
Akbari (2009) 0.64 (0.06, 7.06) 0.39
Huang (2005b) 0.76 (0.36, 1.60) 3.74
McKean (2009) 1.13 (0.65, 1.97) 5.19
Subtotal (I-squared = 0.0%, p = 0.666) 0.96 (0.62, 1.49) 9.32
.
HB
Betti (2011) 1.98 (0.08, 49.64) 0.13
Feng (2008) 1.81 (1.09, 3.02) 4.99
Hall (2007) 2.25 (1.33, 3.82) 4.22
Li (2005) 0.40 (0.04, 3.92) 0.60
Rajaraman (2010) 1.02 (0.42, 2.45) 2.23
Rajaraman (2010) 1.24 (0.39, 3.91) 1.11
Rajaraman (2010) 1.21 (0.26, 5.53) 0.64
Zhang (2010) 0.70 (0.29, 1.70) 2.79
Huang (2010) (Excluded) 0.00
Subtotal (I-squared = 11.0%, p = 0.344) 1.52 (1.14, 2.03) 16.72
.
Overall (I-squared = 3.2%, p = 0.410) 1.32 (1.17, 1.49) 100.00
.00887
1
113

Table S1. Basic information of included studies

Author	Year	Country	Race	Case	Control	Control source	Quality score	Genotyping Assay	Mean age (case/control)	Gender (Males %)
**Agalliu, et al**	**2010**	USA	Caucasian	1,250	1,237	PB	6	SNPlex^{TM} Genotyping system	NA	NA
		USA	African	147	81	PB	6	SNPlex^{TM} Genotyping system	NA	NA
**Akbari, et al**	**2009**	Iran	Asian	196	250	PB/HB	6	iPLEX-MALDI-TOF-MassARRAY	63.6/55.2	50.9/51.0
**Betti, et al**	**2011**	Italy	Caucasian	133	251	PB	8	TaqMan	66.8/61.7	67.0/69.0
		Italy	Caucasian	68	44	HB	8	TaqMan	68.39/68.0	70.0/77.0
**Bye, et al**	**2011**	South Africa	African	346	469	PB	6	TaqMan	59.8/NA	50.8/NA
		South Africa	Mixed	196	423	PB	6	TaqMan	60.5/NA	65.2/NA
**Chae, et al**	**2006**	Korea	Asian	432	432	PB	7	PCR-RFLP	61.6/60.9	81.5/81.5
**Chuang, et al**	**2011**	Mixed	Mixed	1,455	3,160	PB	6	NA	NA	78.9/74.0
**Doecke, et al**	**2008**	Australia	Mixed	566	1,337	PB	8	SequenomiPLEXTM	64.0/61.0	66.0/89.0
**Felini, et al**	**2007**	USA	Caucasian	379	459	PB	7	TaqMan	NA	NA
**Feng, et al**	**2008**	China	Asian	201	201	HB	6	PCR-RFLP	NA	NA
**Gu, et al**	**2009**	USA	Caucasian	214	212	PB	5	NA	NA	NA

**Hall, et al**	**2007**	Mixed	Caucasian	779	1,034	HB	6	TaqMan	NA	87.9/76.7
**Han, et al**	**2006a**	USA	Caucasian	434	1,085	PB	7	TaqMan	NA	NA
**Han, et al**	**2006b**	USA	Caucasian	1,276	1,714	PB	8	TaqMan	NA	NA
**Hu, et al**	**2013**	China	Asian	543	495	PB	8	PCR-RFLP	51.5/50.9	62.1/53.9
**Hu, et al**	**2007**	China	Asian	500	517	PB	8	Illumina SNP genotyping Bead Lab platform	59.3/60.0	77.2/77.4
**Huang, et al**	**2017**	China	Asian	90	90	PB	7	SNaPshot	44.4/49.6	53.3/57.8
**Huang, et al**	**2007**	China	Asian	539	800	PB	7	modified PCR-mismatch amplification	NA	NA
**Huang, et al**	**2010**	China	Asian	176	110	HB	6	DNA sequencing	54.0/55.7	92.0/60.9
**Huang, et al**	**2005a**	Poland	Caucasian	280	387	PB	6	MALDI-TOF/Hme chemistry	NA	66.0/66.0
**Huang, et al**	**2005b**	USA	Mixed	514	754	PB/HB	6	TaqMan/MALDI-TOF	56.0/55.0[a]<br>60.0/58.0[b]<br>65.0/57.0[c]	71.0/70.0[a]<br>79.0/56.0[b]<br>90.0/78.0[c]
**Inoue, et al**	**2003**	Japan	Asian	73	224	PB	6	PCR-SSCP	45.5/46.1	NA
**Kiczmer, et al**	**2018**	Poland	Caucasian	69	239	PB	6	TaqMan	56.1/34.7	71.0/44.6
**Kietthubthew, et al**	**2006**	Thailand	Caucasian	106	164	PB	9	PCR-RFLP	67.1/68.4	72.6/55.5
**Li, et al**	**2005**	China	Asian	167	204	HB	8	PCR-RFLP	63.6/59.2	80.2/84.8

**Liu, et al**	**2002a**	China	Asian	60	100	PB	8	PCR-SSCP/ sequencing	50.7/49.0	67.1/58.9
**Liu, et al**	**2002b**	China	Asian	58	100	PB	8	PCR-SSCP/ sequencing	49.5/50.0	53.0/51.1
**Liu, et al**	**2006**	China	Asian	100	65	PB	6	microarray-based method	57.7/59.1	88.0/78.5
**Liu, et al**	**2009**	USA	Caucasian	369	363	PB	8	SequenomMassARRAYiPLEX platform	NA	56.8/43.6
**Loh, et al**	**2011**	UK	Caucasian	188	1,120	PB	6	genome-wide association scan	64.6/61.9	57.8/51.8
**Lu, et al**	**2006**	China	Asian	191	251	PB	9	PCR-RFLP	61.8/61.4	73.8/72.5
**McKean, et al**	**2009**	USA	Caucasian	998	1,968	PB/HB	6	Multiple methods	56.3/53.6	61.0/51.1
**O'Mara, et al**	**2011**	Australia	Caucasian	1,173	1,099	PB	7	SequenomMassARRAY platform	NA	NA
		Poland	Caucasian	397	406	PB	7	Illumina iSelect Custom Bead Chip	NA	NA
**Palli, et al**	**2010**	Italy	Caucasian	291	537	PB	6	TaqMan	NA	56.4/49.3
**Rajaraman, et al**	**2010**	USA	Caucasian	546	477	HB	5	TaqMan	51.2/49.2[d]<br>54.8/49.2[e]<br>51.7/49.2[f]	54.7/46.1[d]<br>22.4/46.1[e]<br>36.2/46.1[f]
**Ritchey, et al**	**2005**	China	Asian	161	246	PB	8	MALDI-TOF/Hme chemistry	72.2/71.7	NA
**Shah, et al**	**2012**	India	Asian	92	77	PB	8	PCR-RFLP	58.0/51.8	69.5/67.5
**Shen, et al**	**2005**	USA	Caucasian	1,064	1,107	PB	7	FP	NA	NA

**Shen, et al**	**2007**	Australia	Caucasian	555	495	PB	6	TaqMan	NA	NA
**Shi, et al**	**2011**	South Africa	African	303	554	PB	6	SequenomiPLEXTM	42.2/NA	38.4/NA
**Stern, et al**	**2007**	China	Asian	292	1,166	PB	7	TaqMan	61.6/56.5[g]<br>60.9/56.5[h]	52.0/43.0[g]<br>60.9/56.5 [h]
**Tranah, et al**	**2006**	USA	Caucasian	186	2,137	PB[@]	7	TaqMan	NA	NA
		USA	Caucasian	257	429	PB[#]	7	TaqMan	NA	NA
**Wang, et al**	**2006**	USA	Caucasian	1,121	1,163	PB	9	PCR-RFLP	61.4/61.1	52.6/49.4
**Yang, et al**	**2009**	China	Asian	48	352	PB	7	MassARRAY method	NA	58.3/52.3
**Zhang, et al**	**2008**	China	Asian	406	782	PB	8	TaqMan	NA	27.4/38.8[i]<br>59.8/38.8[j]<br>51.1/38.8[k]
**Zhang, et al**	**2010**	USA	Caucasian	721	1,234	HB	8	PCR-RFLP	57.0/57.1	74.9/74.1
**Zienolddiny, et al**	**2006**	Norway	Caucasian	304	363	PB	8	APEX	NA	75.8/76.5

Abbreviations: PB, population-based control; HB, hospital-based control; @, control from Nurses' Health Study (NHS); #, controls from Physicians' Health Study (PHS) cohorts; PCR, polymerase chain reaction; RFLP, restriction fragment-length polymorphism; NA, not available; FP, fluorescence polarization; SNP, single nucleotide polymorphisms; MALDI-TOF, matrix-assisted laser desorption/ ionization time of flight mass spectrometry; hME, homogeneous mass extend; SSCP, single-strand conformation polymorphism; APEX, arrayed primer extension technique.

a, data in Washington; b, data in North Carolina; c, data in Puerto Rico; d, data of Glioma; e, data of Meningioma; f, data of Acoustic neuroma; g, data of cancer in colon; h,

data of cancer in rectum; i, data of gallbladder cancer; j, data of bile duct; k, data of Ampulla of Vater cancer.

Table S2. Data of subgroup analysis under allele T vs. C model

Factor	Subgroup	Sample size			Heterogeneity		Association	
		Study	Case	Control	I^2	*P*	*P*	OR (95% CI)
Race	Caucasian	27	13,158	20,678	18.5%	0.196	0.833	-
	African	3	796	1,104	0.0%	0.752	0.955	-
	Asian	20	4,325	6,562	63.0%	<0.001	0.230	-
Cancer type	Urinary system cancer	4	1,725	1,768	64.7%	0.037	0.192	-
	esophageal cancer	8	2,131	3,907	30.3%	0.186	0.185	-
	lung cancer	5	2,417	2,575	0.0%	0.411	0.404	-
	head and neck cancer	14	5,863	10,581	67.7%	<0.001	0.493	-
	gastric cancer	3	762	1,175	0.0%	0.674	0.429	-
	blood cancer	3	906	1,401	62.3%	0.070	0.694	-
	colorectal cancer	3	735	3,732	0.0%	0.520	0.183	-
	brain cancer	9	2,998	5,030	74.5%	<0.001	0.778	-
	glioma	5	1,735	1,884	83.1%	<0.001	0.637	-
Control source	population-based control	42	16,644	26,788	55.0%	<0.001	0.122	-
	hospital-based control	9	2,658	4,258	2.7%	0.412	0.694	-

Abbreviations: OR, odds ratio; CI, confidence interval.

-, OR (95% CI) data was not provided, when *P* value of association >0.05.

Table S3. Data of subgroup analysis under C/T vs. C/C model

Factor	Subgroup	Sample size			Heterogeneity		Association	
		Study	Case	Control	I^2	*P*	*P*	OR (95% CI)
Race	Caucasian	27	13,158	20,678	20.2%	0.175	0.079	-
	African	3	796	1,104	0.0%	0.989	0.670	-
	Asian	20	4,325	6,562	55.8%	0.001	0.379	-
Cancer type	Urinary system cancer	4	1,725	1,768	72.7%	0.012	0.330	-
	esophageal cancer	8	2,131	3,907	44.7%	0.081	0.896	-
	lung cancer	5	2,417	2,575	0.0%	0.557	0.856	-
	head and neck cancer	14	5,863	10,581	57.0%	0.004	0.059	-
	gastric cancer	3	762	1,175	0.0%	0.788	0.291	-
	blood cancer	3	906	1,401	62.9%	0.067	0.695	-
	colorectal cancer	3	735	3,732	0.0%	0.984	0.041	0.79 (0.64, 0.99)
	brain cancer	9	2,998	5,030	66.2%	0.003	0.461	-
	glioma	5	1,735	1,884	78.1%	0.001	0.960	-
Control source	population-based control	42	16,644	26,788	49.7%	<0.001	0.756	-
	hospital-based control	9	2,658	4,258	12.4%	0.331	0.027	0.86 (0.75, 0.98)

Abbreviations: OR, odds ratio; CI, confidence interval. -, OR (95% CI) data was not provided, when *P* value of association >0.05.

Table S4. Data of subgroup analysis under C/T+T/T vs. C/C model

Factor	Subgroup	Sample size			Heterogeneity		Association	
		Study	Case	Control	I^2	*P*	*P*	OR (95% CI)
Race	Caucasian	27	13,158	20,678	15.3%	0.240	0.355	-
	African	3	796	1,104	0.0%	0.909	0.796	-
	Asian	20	4,325	6,562	59.3%	<0.001	0.282	-
Cancer type	Urinary system cancer	4	1,725	1,768	70.2%	0.018	0.255	-
	esophageal cancer	8	2,131	3,907	33.7%	0.159	0.491	-
	lung cancer	5	2,417	2,575	0.0%	0.500	0.624	-
	head and neck cancer	14	5,863	10,581	62.0%	0.001	0.177	-
	gastric cancer	3	762	1,175	0.0%	0.722	0.334	-
	blood cancer	3	906	1401	64.2%	0.061	0.686	-
	colorectal cancer	3	735	3,732	0.0%	0.845	0.077	-
	brain cancer	9	2,998	5,030	71.8%	<0.001	0.605	-
	glioma	5	1,735	1,884	81.7%	<0.001	0.793	-
Control source	population-based control	42	16,644	26,788	52.9%	<0.001	0.347	-
	hospital-based control	9	2,658	4,258	0.0%	0.556	0.114	-

Abbreviations: OR, odds ratio; CI, confidence interval. -, OR (95% CI) data was not provided, when *P* value of association >0.05.

Table S5. Data of subgroup analysis under carrier T vs. C model

Factor	Subgroup	Sample size			Heterogeneity		Association	
		Study	Case	Control	I^2	*P*	*P*	OR (95% CI)
Race	Caucasian	27	13,158	20,678	0.0%	0.820	0.530	-
	African	3	796	1,104	0.0%	0.916	0.877	-
	Asian	20	4,325	6,562	40.9%	0.030	0.159	-
Cancer type	Urinary system cancer	4	1,725	1,768	55.6%	0.080	0.658	-
	esophageal cancer	8	2,131	3,907	0.0%	0.563	0.348	-
	lung cancer	5	2,417	2,575	0.0%	0.718	0.676	-
	head and neck cancer	14	5,863	10,581	43.5%	0.041	0.020	0.92 (0.85, 0.99)
	gastric cancer	3	762	1,175	0.0%	0.820	0.483	-
	blood cancer	3	906	1,401	44.6%	0.164	0.853	-
	colorectal cancer	3	735	3,732	0.0%	0.820	0.160	-
	brain cancer	9	2,998	5,030	58.1%	0.014	0.694	-
	glioma	5	1,735	1,884	72.9%	0.005	0.259	-
Control source	population-based control	42	16,644	26,788	28.4%	0.047	0.723	-
	hospital-based control	9	2,658	4,258	0.0%	0.771	0.404	-

Abbreviations: OR, odds ratio; CI, confidence interval. -, OR (95% CI) data was not provided, when *P* value of association >0.05.

Table S6. Publication bias result

Genetic models	Study (number)	Begg's Test		Egger's test	
		P	**z**	***P***	**t**
**allele T vs. C**	54	0.189	1.31	0.118	1.59
**T/T vs. C/C**	50	0.763	0.30	0.651	-0.46
**T/T vs. C/C+C/T**	50	0.688	0.40	0.440	-0.78
**C/T vs. C/C**	54	0.303	1.03	0.117	1.59
**C/T+T/T vs. C/C**	54	0.144	1.46	0.084	1.76
**carrier T vs. C**	54	0.179	1.34	0.087	1.75
